# Deep eutectic solvents for green extraction and separation of bioactive compounds from traditional Chinese medicines

**DOI:** 10.1186/s13020-026-01325-z

**Published:** 2026-01-15

**Authors:** Ahmad Ali, Ruirui Li, Ruiliang Zhu, Subhan Mahmood, Qianfeng Chen, Shun Yao

**Affiliations:** 1 School of Chemical Engineering, Sichuan University, Chengdu, 610065 China; 2 College of Pharmaceutical Sciences, Southwest University, Chongqing, 400715 China

**Keywords:** Deep Eutectic Solvents (DESs), Traditional Chinese Medicine (TCM), Extraction, Separation, Bioactive compounds, Environmental-Health-Safety (EHS)

## Abstract

**Background:**

Traditional Chinese Medicines (TCM) has long relied on bioactive compounds derived from natural sources, but conventional extraction and separation methods often involve violate/hazardous organic solvents, posing environmental and health risks. Deep Eutectic Solvents (DESs) have emerged as a sustainable alternative, offering tunable physicochemical properties, biodegradability, and enhanced extraction efficiency for TCM constituents such as alkaloids, flavonoids, and polysaccharides.

**Methods:**

This review comprehensively summarizes the synergistic integration of DESs with TCM, highlighting their applications in green extraction, purification, and stabilization of bioactive compounds. It investigates a series of separation techniques, including liquid/solid–liquid (micro) extraction, chromatographic systems and others, where DESs enhance efficiency and recyclability. Environmental-Health-Safety (EHS) analyses, such as life cycle assessments and related tools, are also discussed.

**Results:**

DESs demonstrate superior performance in preserving heat-sensitive compounds, improving solubility, and enabling selective extraction as well as isolation while aligning with green chemistry principles. However, challenges such as high viscosity, scalability, and toxicological assessments remain. Despite these limitations, DESs show significant eco-friendly potential, and future opportunities in policy support and AI-driven design could further advance their role in modernizing TCM for safer, more efficient, and sustainable therapeutic development.

## Introduction

For millennia, TCM has served as a cornerstone of healthcare, utilizing intricate formulations of bioactive compounds sourced from herbs, minerals, and animal products to treat a wide array of ailments [1]. During the modernization process of TCM, the trend of interdisciplinary development is becoming more and more obvious. It has formed a consensus that TCM requires support from different disciplines to achieve progress. In recent years, the global resurgence of interest in natural and holistic therapies has spurred extensive research into optimizing TCM extraction, purification, and formulation processes to enhance efficacy, safety, and sustainability [2]. TCM usually have a low content of active ingredients and a complex system composition, including both large and small molecules, polar and non-polar substances, target components and impurities. Extraction refers to the process of using appropriate solvents or (chemical, physical, biological) methods to obtain target components from natural raw materials, reflecting the migration and transfer processes of related substances between different environments. The fundamental principle of extraction is to maximize the dissolution of target compounds while minimizing the co-extraction of undesired constituents; such an outcome reflects high selectivity [3]. The extraction process should not affect its structure and should be conducive to simplifying the post-processing process, and potential extractants often play the key role. Traditional extraction methods for TCM are reliable and easy to industrialize, but they also have some common problems such as low extraction efficiency, more solvent consumption, high impurity content, and long production cycles [4]. Currently, the latest progress in TCM extraction is reflected in the following aspects:

- continuous research and development of new extraction solvents, such as innovative green solvents represented by ILs and DESs, bio based solvents (biodiesel/carbohydrates/lignin, etc.), natural terpenoid solvents (such as limonene/eucalyptus oil/turpentine, etc.), and polymer solvents (such as polyethylene glycol/polypropylene glycol/polyether, etc.); these new solvents often achieve an acceptable balance between efficiency, friendliness, and economy; and their recyclable characteristics can further reduce their usage costs.
- The optimization of solvent extraction is being continuously promoted by upgraded computational chemistry, ML, and even AI technologies, greatly reducing the time and experimental work required for the “trial-and-error method” in the initial stage of establishing process conditions, as well as predicting and guiding the synthesis of optimal solvents. The existing universal quasi-chemical functional-group activity coefficient models (UNIFAC), quantum mechanics (QM), quantitative structure–activity relationship (QSAR), density functional theory (DFT) based thermodynamic property prediction model (COSMO), artificial neural network (ANN), molecular dynamics (MD), and docking calculation all demonstrate their respective advantages [5]. With the emergence of more and more (commercialized) software and related databases, the convenience enjoyed by researchers in the selection of extractants is becoming increasingly prominent.
- More applications and coupling of new physical fields are appearing in current extraction ways; for instance, the integration of electric field, magnetic field, and light energy field with existing processes urgently needs to be promoted and should step out of the laboratory and into actual production as soon as possible. On the one hand, the physicochemical properties of solvents in these physical fields are different from conventional states and can be utilized; on the other hand, the quality efficiency of the whole system can also be improved after receiving different types of energy.
- The appearance of more mass transfer-enhanced methods is another important driving force, such as using mechanochemical assisted extraction. They utilize high-strength mechanical force to fully grind, shear, and extrude raw materials into ultrafine powder state, increasing the specific surface area of solid particles, promoting cell wall breaking and physicochemical property changes, thereby effectively promoting the dissolution of target components. Different types of solid/liquid additives can also be added to achieve selective extraction of target components.
- The emergence and promotion of solvent-free extraction process cannot be ignored either, which was first and most commonly used in the extraction process of natural oils (including volatile oils/essential oils) or animal/plant juices, often using wall breaking methods such as rolling, squeezing, shearing, shaking, stirring, microwave, or adsorption to obtain the target substance [6]. Due to the absence of any solvents, the advantages of this method are very obvious, but the extraction targets are relatively limited and need to be expanded.

Extraction represents the initial stage of processing, serving to separate the complex chemical mixture present in crude extracts from the raw plant matrix. When further research, development, and production of TCM are needed, the subsequent enrichment and separation process becomes particularly important, and the time and effort invested in the corresponding steps are also at the forefront [7]. If the separation efficiency can be effectively improved, it is of great significance to accelerate the overall research and development progress; continuous innovation is urgently needed for unit operations involving solidified organic solvents and conventional methods [8]. The conventional enrichment and separation methods mainly include fractionation, liquid–liquid extraction, solid–liquid extraction, precipitation, salting out, dialysis, crystallization [9]. Molecular distillation, multi-component distillation, special (azeotropic/extraction/salt addition) distillation techniques, aqueous two-phase and multiphase extraction, adsorption and ion exchange, membrane separation, pervaporation, and various electrophoresis/chromatography methods (including corresponding instruments and equipment’s) that form a series of families are also widely used in current basic research and practical production. Especially in the process of fine separation, one-dimensional/multi-dimensional/multi-channel chromatography technology is still the mainstream with silica, diatomite, alumina, cellulose, activated carbon, molecular sieve and various polymer resins or gels as the main media. Their application is relatively mature and their performance is relatively stable. At the same time, their economy can meet the requirements of laboratories and production enterprises [10]. In theory, the aforementioned green solvents can be combined with these techniques without any obstacle. In addition, various emerging online combination technologies (such as chromatography-chromatography, chromatography-spectroscopy, chromatography-bioactivity assay, chromatography-spectroscopy-bioactivity assay, etc.) can make the entire process more continuous and functionally comprehensive. The highly automated, modular, and intelligent integrated devices are strongly driving the continuous updating and further promotion of the existing separation methods. What is even more exciting is that robots have gradually become a reality in replacing humans for lengthy, complex, and highly demanding separation tasks that require high levels of operation and programming [11].

At present, there are a lot of researches on the applications of various extraction and separation techniques in TCM, and the original innovation in methodology is not easy. Once new commercial equipment’s are introduced, their powerful functions often attract widespread interest from academia and industry; but their price and usage cost are also significantly high, and the research and development cycle are relatively long [12]. Where is the source of practical innovative technology when there are no updated instruments? As TCM R&D personnel who often deal with extraction and separation, they are well aware of own needs, object characteristics, and the advantages and disadvantages of commonly used technologies. Based on this solid foundation, they also can actively participate in the development of new methods, from passive users to active developers. Apart from breaking through existing efficiency, is there any room for improvement in the friendliness and greenness of current operational processes [13]? These are all thought-provoking questions. In the opinions of our authors, the system and medium are two aspects that require special attention, which are relatively easy to innovate. The innovative technology established from these focuses more on energy conservation and emission reduction (low-carbon), the decreased discharge of toxic and harmful substances, as well as efficient and convenient use. In summary, a new direction is the innovative development of green, energy-saving, and environmentally friendly methods based on existing foundations. DESs is such a featured green and friendly medium; its structure can be designed, and its functions are quite comprehensive. The DES-based methods are expected to fully align with the famous twelve golden principles of green chemistry. Currently, its application in the field of TCM extraction and separation is increasing and receiving much attention, with superior performance compared to traditional solvents.

### Bibliometric analysis on DES-involved studies of natural products

**Fig. 1:**
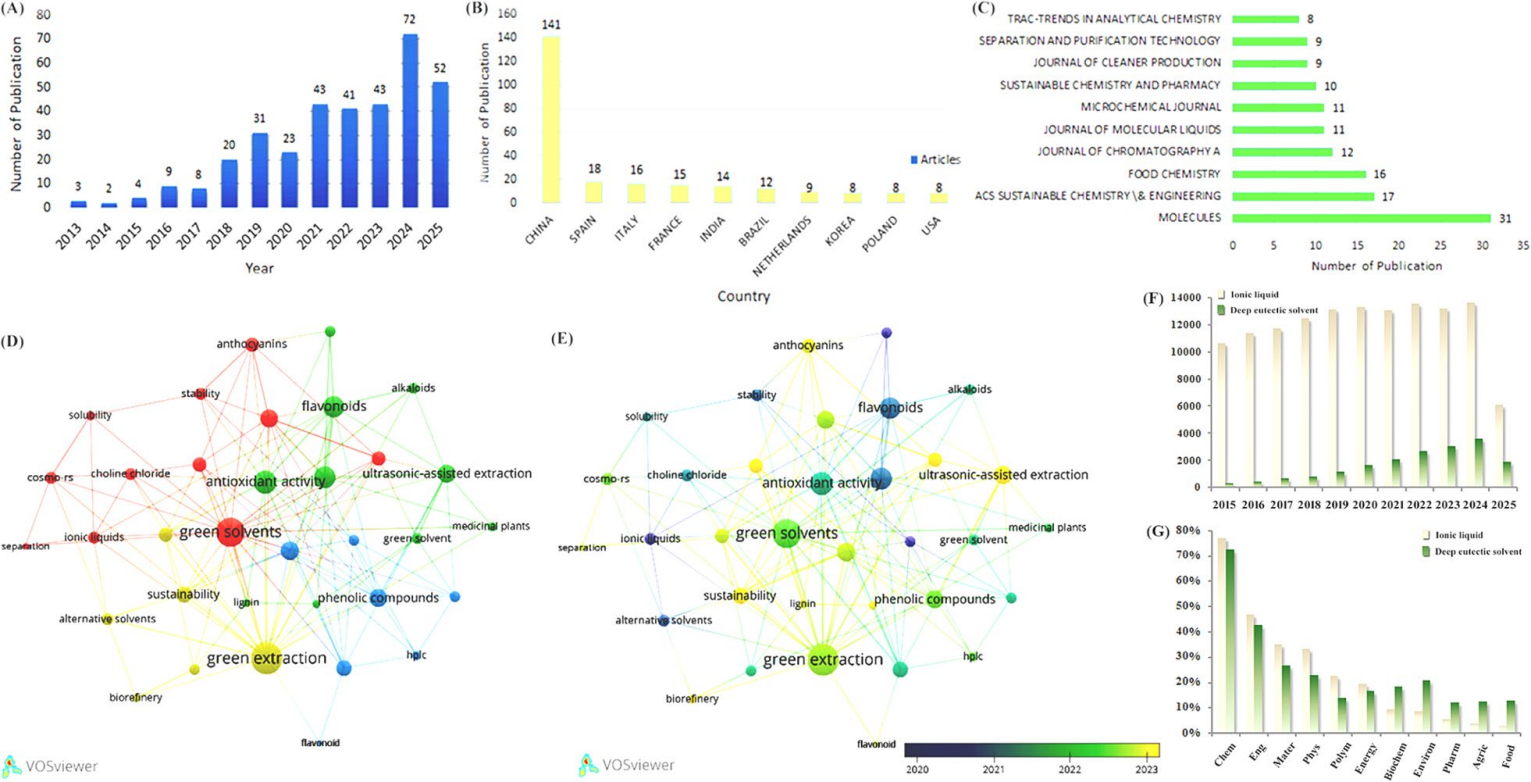
**A** Number of publications per year from 2013 to 2025; **B** number of publications in different countries; **C** number of publications of different journals; **D** network visualization map of DES-based studies on natural bioactive constituents; **E** overlay visualization map of des-based studies on natural bioactive constituents; **F** the number of articles on ILs and DESs and; **G** Percentage distribution of DES and IL publications across major research subjects (2015–2025). Percentages are calculated based on Web of Science research areas; multi-disciplinary articles are counted in all relevant categories

This section employs bibliometric analysis to quantitatively map the research landscape of DESs applied in the extraction and separation of natural bioactive products. By analyzing publication trends, collaborative networks, and thematic hotspots, we aim to identify the evolution of this field, key contributors, and emerging frontiers. The analysis is based on publications retrieved using the core keywords: “DESs,” “natural product,” and “extraction/separation,” providing a focused perspective on DESs-driven green technologies for bioactive compounds. Data were collected from the Web of Science Core Collection (2013–2025) using the search query: TS = ((*DESs*) AND (“*natural product*” or “*bioactive compound*”) AND (“*extract*” or “*separation*”)). Document types are refined to “Article” and “Review article”. On Web of Science^®^ (Clarivate Analytics), 351 papers collected (Research articles: 255 and Review articles: 96). The whole analyses were performed using bibliometric R-package, with visualizations generated via VOS viewer for co-occurrence networks. Between 2013 and 2025, a total of 351 papers investigated natural bioactive constituents using DESs. Figure 1A reveals a significant increase in annual publication volume, climbing dramatically from 3 in 2015 to 72 in 2024, demonstrating an overall exponential growth trend. Figure 1B indicates China contributed disproportionately more publications than any other countries, accounting for 40.1% of the total. Spain ranked second (5.1%), followed by Italy (4.5%). Notably, countries with fewer than 8 publications are omitted from this figure. Figure 1C identifies the top three source journals: Molecules (Impact Factor 2023: 4.6), ACS Sustainable Chemistry & Engineering (Impact Factor 2023: 7.3), and Food Chemistry (Impact Factor 2023: 9.8). The most cited paper within this dataset examined the solubilizing effects of natural deep eutectic solvents (NADES) on poorly soluble biomolecules. This foundational work revealed promising novel solvents applicable to drug extraction and food processing applications.

Bibliometric studies rely heavily on analyzing frequently appearing keywords. Examining how keywords appear together reveals existing research themes and highlights emerging topics requiring further investigation. This field uses the author keywords displayed in Fig. 1D representing the thirty-six most frequent terminology. Each cluster in this network map contains unique keywords shown by various colors, while the clusters emerged through keyword (co-occurrence) relationships across multiple documents to show related pairs. Each cluster in the visual representation reflects the total link strength of individual keywords through circle size scale indicators. The distance between the mentioned terms within the visualization reveals the strength of the relationship. Moreover, Fig. 1D demonstrates that keyword co-occurrence analysis identified four distinct thematic clusters. The red and green clusters feature keywords centered around “green solvents” and “green extraction,” reflecting a focus on solvent design for environmentally benign separation processes. The green and blue clusters primarily encompass keywords related to specific bioactive targets; here, “flavonoids,” “phenolic compounds,” and “alkaloids” exhibit strong linkages to “medicinal plants” and “antioxidant activity,” highlighting key natural products driving extraction research. Figure 1E reveals a significant recent surge in the frequency of the keywords “green solvents” and “green extraction,” indicating growing research emphasis on solvent safety and environmental sustainability/recyclability. Additionally, “ultrasound-assisted extraction” emerged as a dominant technique with extensive cross-cluster connections.

Figure 1F and G summarize the number of articles published between 2015 and 2025 on “DESs” and their distribution across various fields (data obtained from Web of Science^®^ (Clarivate Analytics) Core Collection search for “deep eutectic solvent,” with “ionic liquid” included for comparison). Although DESs started much later than ILs (first reported in 2003 versus early twentieth century for ILs) and lag behind in terms of publication numbers, they exhibit significant application value and potential in chemistry, materials science, engineering, agriculture, food, pharmaceuticals and so on, indicating broad development prospects. The relatively smaller number of articles on DESs reflects its later emergence and thus a greater remaining research space. Notably, the article percentage in subjects of pharmacy, agriculture and food on DESs is higher than that on ILs, reflecting the ideal biocompatibility and strong relevance of the former to these fields. According to above results, it can be concluded that there is great potential for their use in the field of Chinese medicines. Figure 1F & G presents the distribution of publications related to DESs and ILs across major research domains during 2015–2025. The proportion for each subject was determined using Eq. (1).

1 $$Percentage in Subject X=\frac{Number of publications in Subject X}{Total publications across all subjects}\times 100\%$$

Subject categories were defined according to the Web of Science research areas and include Chemistry (physical, analytical, applied, and multidisciplinary chemistry), Materials Science (materials characterization, biomaterials, and nanomaterials), Engineering (chemical, environmental, and biomedical engineering), Agriculture & Food Science (agricultural engineering, food science, and nutrition), Pharmacology & Pharmacy (drug discovery, pharmaceutical technology, and pharmacology), Biochemistry & Molecular Biology (bioactive compounds, enzymology, and metabolomics). Publications assigned to multiple subject areas were counted in each relevant category. This analysis underscores the interdisciplinary nature of DES research, particularly its pronounced representation in fields associated with biocompatibility and natural product applications.

To highlight the growing relevance of DESs in natural product research, we conducted a bibliometric analysis of publications from 2015–2025. This analysis underscores their interdisciplinary applications and increasing prominence in pharmaceutical and agricultural fields, thereby justifying their integration into TCM extraction and separation studies.

**Scheme 1:**
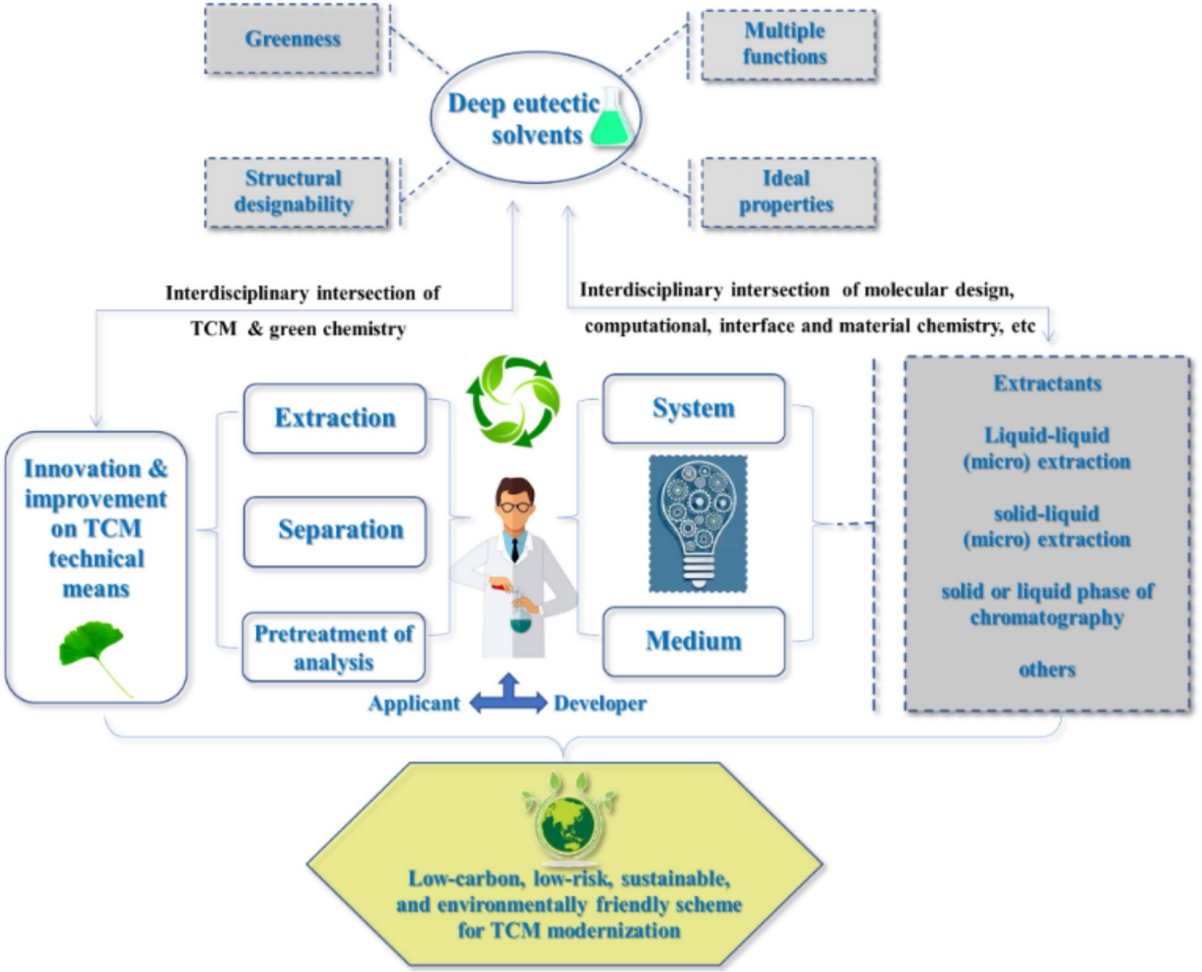
The whole original intention, purpose, and contents of this review

Under above background, this review provides a detailed analysis of the synergistic integration of TCM and DESs, emphasizing their transformative role in sustainable extraction, enrichment and separation, bioactive compound stabilization, and so on [14]. By evaluating recent breakthroughs, ongoing challenges, and future directions, we aim to elucidate the pivotal role of DESs in advancing green chemistry-driven modernization of TCM, paving the way for safer, more efficient, and environmentally responsible therapeutic development. In order to make readers clear, here Scheme 1 is used to depict the whole original intention, purpose, and contents of each section in this review.

### DESs and their main properties related with TCM

#### Overview of DESs

The eutectic phenomenon refers to the formation of a mixture with a melting point significantly lower than that of any individual component when two or more pure substances are combined at a specific molar ratio. The term “deep” in DESs emphasizes that this melting point depression is substantial, often resulting in a liquid state at or near room temperature, far below the melting points of the separate constituents. This behavior arises from strong, non-ideal intermolecular interactions primarily extensive hydrogen bonding networks between HBA and HBD. Consequently, DESs deviate considerably from ideal thermodynamic mixing models. It is crucial to distinguish DESs from simple eutectic mixtures: not all eutectic solvents qualify as DESs. DESs specifically refer to those mixtures where the pronounced melting point depression is driven by complex, multi-component interactions, and they are typically liquid over a wide temperature range. Importantly, DESs are mixtures of molecular or ionic components that retain their individual chemical identities, rather than new pure compounds. This contrasts with many IL, which are often discrete, pure ionic species. First reported by Abbott et al. in 2003 [6], DESs have emerged as a distinct class of green solvents. They not only share certain advantageous properties with IL such as low volatility, high solvation capacity, and structural tunability but are also generally composed of safer, lower-cost, and more readily available starting materials. Their preparation typically involves simple heating and stirring of the components, usually without forming new covalent bonds, making the synthesis straightforward and often yielding high-purity liquid products. Due to these merits, DESs represent promising and sustainable alternatives to traditional organic solvents and even to some IL.

#### Composition and classification of DESs

DESs are mixtures formed at specific molar ratios between HBA and HBD through intermolecular interactions, primarily hydrogen bonding. The melting point of the resulting mixture is lower than that of any individual component. No chemical reactions occur between the components and no new covalent bonds are formed, and neutral ligands may be present in the system. Different types and molar ratios of HBA and HBD yield different DESs. A classic example is a 1:2 molar ratio of choline chlorides (HBA) and urea (HBD). A DESs can consist of more than two components, including three or more. Researchers have systematically summarized the types of HBA and HBD that can form DESs; the former often includes quaternary ammonium salts (e.g., choline chloride), quaternary phosphonium salts, zwitterions (e.g., betaine), and the latter usually includes amides, amines, carboxylic acids, thiourea, polyols, amino acids, sugars, etc. especially, many bioactive compounds in traditional TCM can be applied as HBA and HBD. In addition to these classic components, water molecules with known stoichiometric ratios (including crystal water) can also serve as part of certain DESs (distinct from exogenous moisture introduced by hygroscopicity). With the continuous expansion and deepening of research, the types of DESs have become increasingly diverse, and their classification methods have correspondingly multiplied (see Table 1) [15]. Overall, each type of DESs mentioned below has successful application examples in the field of TCM.

**Table 1:** Classification of DESs in various ways

No	Classified ways	Types	Composition	Notes
1	Component types	Type I	Cat⁺X⁻·zMClₓ	M = Zn, Fe, Sn, Al, Ga, In
		Type II	Cat⁺X⁻·zMClₓ·yH₂O	M = Cr, Co, Cu, Ni, Fe
		Type III	Cat⁺X⁻·zRZ	Z = CONH_2_, COOH, OH
		Type IV	MClₓ + RZ = MClₓ⁻^1^⁺·RZ + MClₓ⁺^1^⁻	M = Al, Zn; Z = CONH_2_, OH
		Type V	A + B = A∙B	Both A and B are non-ionic
2	Ionic compound composition	Inorganic type	Cations + anions + molecules	Inorganic salts + organic molecules
		Organic type		Organic salts + organic molecules
3	Water miscibility	Hydrophilic type	Hydrophilic molecules	e.g., choline chloride-based DESs
		Hydrophobic type	Hydrophobic/hydrophilic mixtures	e.g., menthol + long-chain acids
4	Component number	Binary type	A∙B	Most common
		Ternary type	A∙B∙C	Common
		Polybasic type	A∙B∙∙∙Z	Rare
5	Component source	Conventional type		Synthetic or common chemicals
		NADES type		Natural compounds only
		API-DES type		Active pharmaceutical ingredients only
6	Magnetic property	Non-magnetic type		No magnetic components
		Magnetic type		Contains magnetic components
7	Supramolecular property	Conventional type		No supramolecular components
		Supramolecular type		Contains supramolecular components

#### Computer-aided design and preparation of DESs

**Fig. 2:**
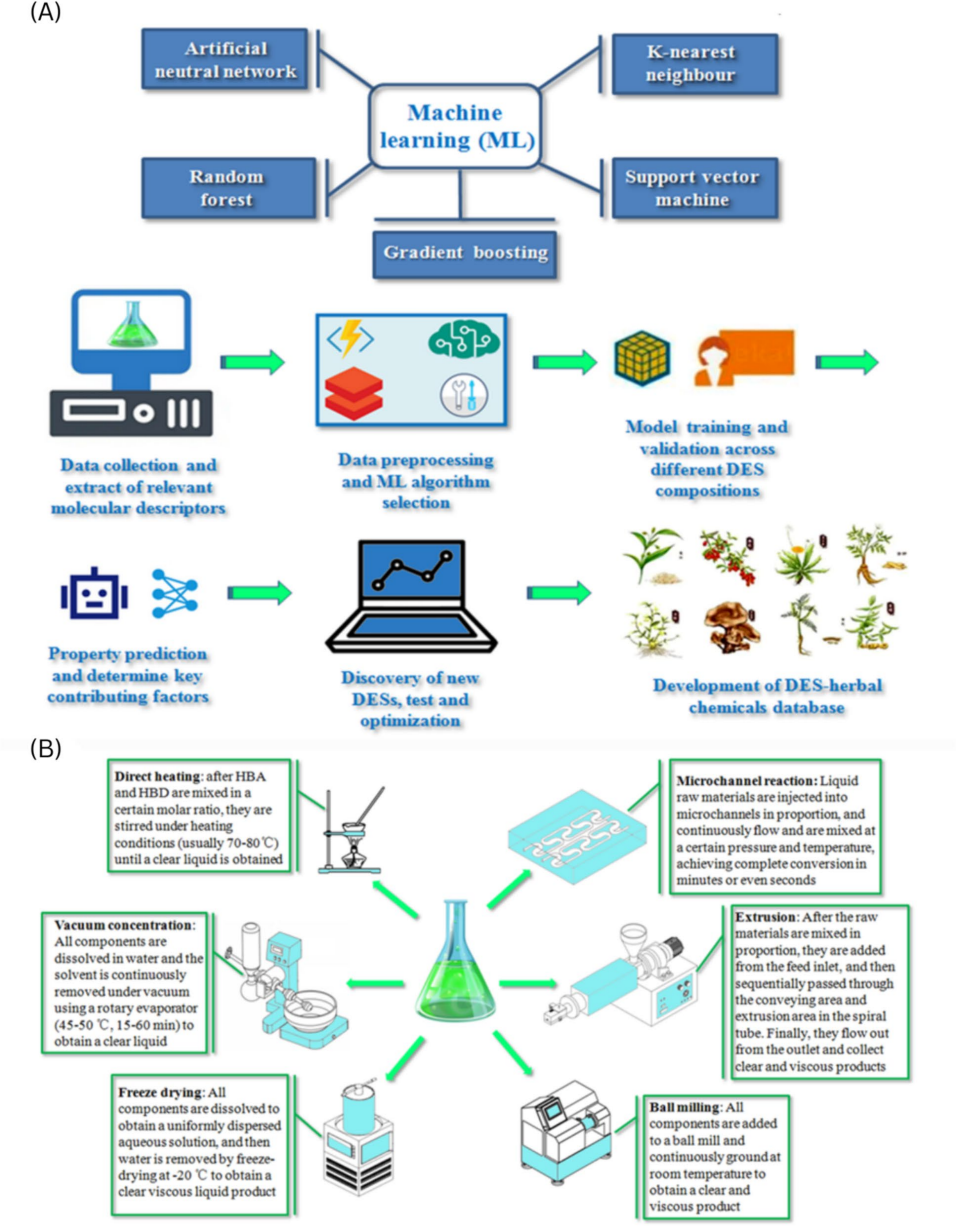
**A** ML methods and steps for DESs studies; **B** preparation ways of DESs

Researchers in this field are often familiar with the components in TCM, but unfamiliar with selecting DESs that match them. At present, there is no mature and comprehensive free or commercial database that provides screening guidance (currently being established by the authors). In this situation, molecular simulation, ML, and AI will effectively improve the screening efficiency of the “trial and error method” based on existing literatures. The structure of DESs can be designed based on the objectives of specific tasks, and their properties can also be predicted through key structure–activity relationships. When applying MD methods to explore the structure and intermolecular interactions between choline chloride and urea, it was found that its formation and melting point decrease were mainly due to the strong hydrogen bonds formed between the anions and the hydroxyl groups of HBD, which has been experimentally confirmed. As the concentration of urea decreased, the hydrogen bond interaction weakened [16]. In recent years, the rise of quantum chemistry has provided a more powerful theory for molecular simulation, and the Conductor-like screening model for real solvents (COSMO-RS) can predict a series of physicochemical properties of DESs based on the phase equilibrium in multi-component mixtures, which has become a powerful tool for screening and designing DESs. Moreover, machine learning algorithms have made the analysis of vast datasets faster, which can be used to predict solubility, density, viscosity, melting temperature, heat capacity, surface tension, and electrical conductivity of DESs (related typical methods and steps can be found in Fig. 2A. There have been 37 articles investigating the prediction of DESs properties using AI tools from 2012 to 2025. The reinforcement learning and generative models validated the potential for independently forecasting innovative DESs compositions with task-specific capabilities [17–19].

The preparation process of DESs is very simple and usually one-step; related components are mixed in a certain molar ratio to prepare the expected products under different conditions. This is very friendly and convenient for professionals in the field of TCM. No chemical reactions occur during the preparation process, and the atomic utilization rate can reach up to 100%. The product often does not require purification. Sometimes adding a small amount of water can reduce the preparation time, temperature, and viscosity of DESs. At present, there are mainly six preparation methods as shown in Fig. 2B. Among them, direct heating is very simple and does not rely on complex equipment; however, the local high temperature can result in possible production of volatile substances and oxidation of phenolic substances [5]. Vacuum evaporation requires a lower heating temperature and is in a vacuum environment, which is beneficial for those unstable DESs components. At the same time, the eutectic products usually contain more water [4]. Freeze drying method can remove more water than vacuum evaporation, but it needs longer duration, more energy consumption [20]. Mechanical methods are usually carried out at room temperature with a simple process, which are suitable for DESs containing thermally unstable components. On the other hand, they require special equipment’s [21, 22]. The microchannel method has heat/mass transfer characteristics that are superior to traditional chemical equipment by 1–3 orders of magnitude, making it a powerful tool for “process enhancement”. But it is not suitable for processing raw materials containing solids. As a whole, researchers can make decision based on their desired yield, purity, preparation efficiency, hardware conditions, and cost-effectiveness.

#### Key properties of DESs for extraction and separation

Polarity is a key property that characterizes the solubility of green solvents in substances. The greater the polarity, the stronger the solubility of polar compounds. The polarity of eutectic solvents can be characterized by the empirical polarity parameter. By adding specific probes (such as Reichardt’s Dye) and measuring the maximum absorbance wavelength (λ_max_) of the dye probe through UV visible spectroscopy, the polarity empirical parameter of the DES can be obtained [23]. The polarity of most eutectic solvents is related to the strength of the HBD ability to form hydrogen bonds. Generally speaking, the stronger the ability of HBD to form hydrogen bonds, the stronger the polarity of the DESs. For example, the DESs formed by choline chloride and glycerol, ethylene glycol, or urea in a molar ratio of 1:2 has a polarity order of choline chloride glycerol > choline chloride ethylene glycol > choline chloride urea, which is mainly related to the number of hydroxyl groups in the HBD. In addition, DESs containing glycerol or ethylene glycol (with hydroxyl groups) has a higher polarity than DESs containing malonic acid or urea (with carboxyl or amide groups). Therefore, their polarity can be fine-tuned by selecting the donor groups in HBD and corresponding ions.

The viscosity unit of DESs is usually measured in terms of Pico pascals (cP) or millipascals per second (MPa). For chloride-malonic acid (1:1) at 25 °C (721 MPa) can reach 800 times that of water viscosity (0.89 MPa), but the viscosity of the DESs composed of choline chloride and urea in a molar ratio of 1:2 (169 MPa) is only over 100 times that of water viscosity. From this, it can be seen that there may be significant differences in viscosity between DESs with different compositions, but their viscosity is mostly in the range of 10–5000 MPa at room temperature and pressure. Generally, those with viscosity below 500 MPa is referred to as low-viscosity DESs. The main internal factors affecting the viscosity are the types and ratios of HBA and HBD, while the external factor is temperature (which can be described using Arrhenius’ logarithmic formula [24]. When DESs are designed, it is suggested to choose HBD of the same type with lower viscosity to obtain low-viscosity products. The addition of water also generally reduces their viscosity and provides convenience for their subsequent applications. This is because there are a large number of hydrogen bonds in water molecules that alter the original hydrogen bond structure of DESs.

There is a significant relationship between conductivity and viscosity, with higher viscosity resulting in lower conductivity. Therefore, most DESs have low conductivity, generally less than 1 mS/cm at 25 °C. Only some low-viscosity DESs (such as those containing ethylene glycol) exhibit higher conductivity. Generally, the conductivity of DESs increases with the increase of salt content in the composition, but this does not apply to all DESs; because their conductivity is not only related to salt concentration, but also to the type of salts and HBD. For example, the conductivity of tetrabutylammonium chloride-ethylene glycol decreases with increasing concentration of tetrabutylammonium chloride. The conductivity of some DESs also shows a trend of first increasing and then decreasing with increasing salt concentration. Finally, the conductivity of DESs generally increases with temperature, and the kinetic energy generated by heating increases the frequency of intermolecular collisions, resulting in a decrease in intermolecular forces and viscosity [25]. Similar to high-temperature molten salts and ILs, DESs have a higher surface tension. When external conditions are the same, the surface tension of a liquid with a constant composition is constant. As a reference, the common surface tension of water at 25 °C is 75.8 mN/m. The surface tension of DESs is mainly related to factors such as intermolecular forces, cation types, and temperature [26]. In most cases, the surface tension is higher than that of common organic solvents, but lower than that of water. The experimental results indicate that increasing the cationic alkyl chain length leads to higher surface tension, and the surface tension of glucose based DESs is higher than that of carboxylic acid based DESs. At present, most DESs have a surface tension higher than that of molecular solvents (22.4 mN/m and 15.5 mN/m for ethanol and n-hexane at 2 °C), and are close to that of high-temperature molten salts (77.3 mN/m for KBr at 900 °C).

#### NADES and their relevance to TCM

NADES represent a unique class of green solvents formed entirely from natural primary metabolites such as sugars, organic acids, amino acids, and choline derivatives. Unlike conventional DES, which may involve synthetic or semi-synthetic components, NADES are composed of biologically compatible molecules that are abundant in living systems. Their formation relies on hydrogen-bond interactions that yield liquids with tunable physicochemical properties, making them highly versatile for extraction and formulation purposes [27]. NADES are closely aligned with the principles of green chemistry. Their components are biodegradable, non-toxic, and renewable, offering significant advantages over conventional organic solvents. The low environmental footprint of NADES, combined with their intrinsic bio-compatibility, makes them particularly suitable for applications in traditional Chinese medicine (TCM), where preservation of natural bioactivity and safety for human use are paramount. Their mild preparation conditions and absence of volatile organic compounds further reinforce their sustainability credentials. Recent studies have demonstrated that NADES outperform conventional DES in the extraction of heat-sensitive and polar bioactive compounds from medicinal plants. For example, NADES systems based on sugar organic acid combinations have shown superior efficiency in extracting flavonoids, alkaloids, and phenolic compounds, while maintaining their structural integrity and pharmacological activity. This is particularly relevant in TCM, where the therapeutic efficacy of herbal formulations depends on the preservation of delicate bioactive molecules. Moreover, NADES can enhance solubility and stability of compounds that are otherwise poorly extracted by traditional solvents, thereby broadening the spectrum of accessible phytochemicals.

### DES‑based extraction of bioactive compounds from Chinese medicines

#### Conventional extraction modes assisted by DESs

Crude extraction is a fundamental step in the processing of Chinese medicines, aiming to isolate bioactive compounds such as alkaloids, flavonoids, polyphenols, and polysaccharides from herbal matrices. Traditional extraction methods, including maceration, Soxhlet extraction, and reflux with water or organic solvents (e.g., ethanol, methanol), often suffer from inefficiency, high energy consumption, and environmental toxicity [28]. DESs have emerged as a sustainable alternative due to their ability to enhance extraction yields while minimizing solvent waste. By virtue of adjustable performance through the flexible combination of HBD and HBA, DESs exhibit high solvation power, low volatility, and tunable polarity, making them ideal for dissolving diverse phytochemicals [29].

**Fig. 3:**
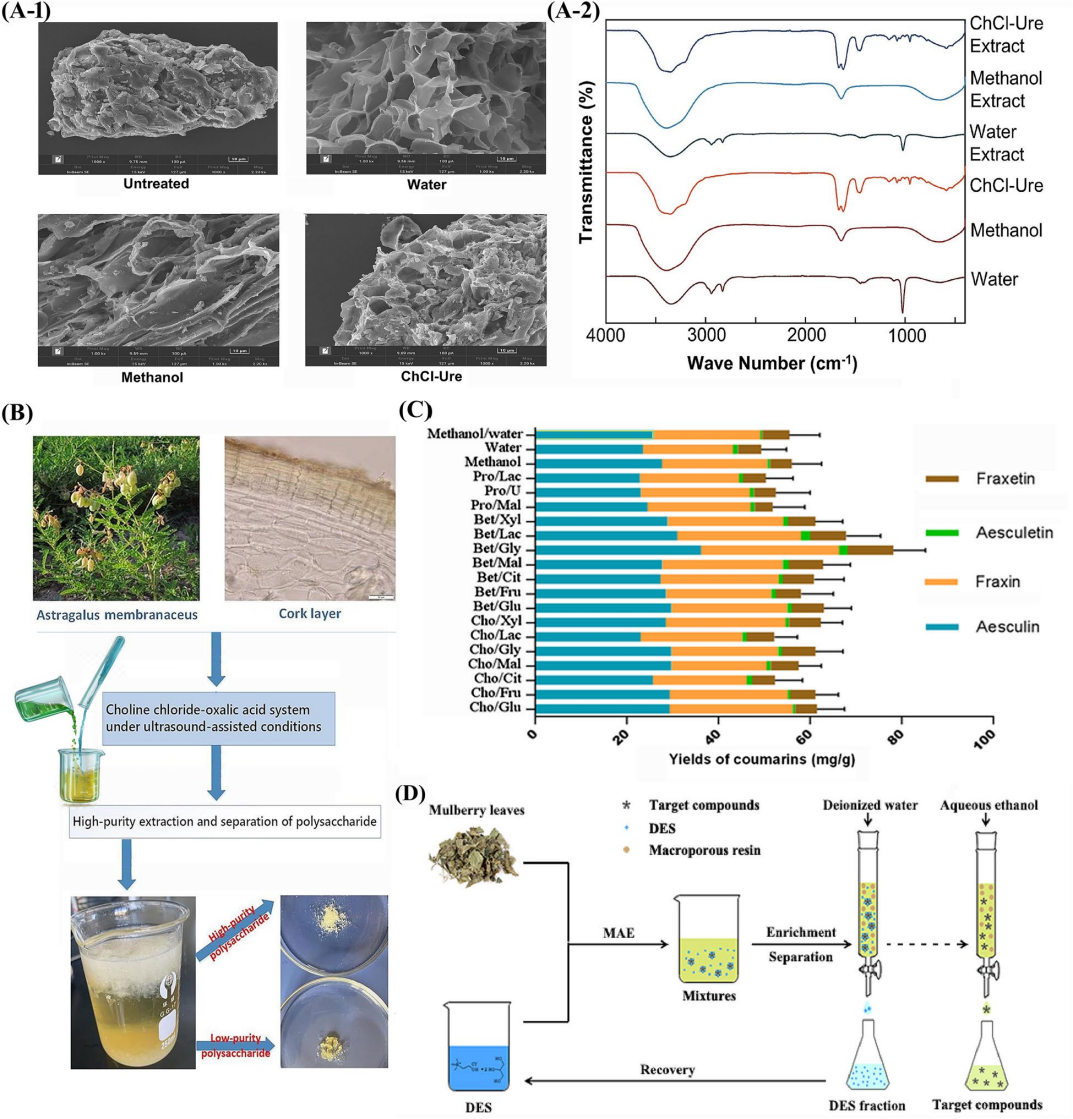
**A**-**1** *Panax ginseng* powders untreated or treated by different solvents and (**A**-**2**) FTIR analysis of different solvents before and after extraction [31]; **B** DESs used for the high-purity extraction and separation of polysaccharides from *Astragalus membranaceus* [36]; **C** comparison of DESs extraction method with conventional solvents for coumarins from *Cortex Fraxini* [51]; **D** scheme of enhanced phenolic compounds extraction from mulberry by DESs [59]

Studies have demonstrated that DESs significantly improve the extraction efficiency of key bioactive compounds from Chinese herbs. For example, a choline chloride-glycerol of DES was found to extract higher amounts of flavonoids from *Scutellaria baicalensis* (Huang Qin) compared to conventional ethanol extraction [30]. Similarly, a lactic acid–glucose-based DESs enhanced the recovery of ginsenosides from *Panax ginseng* (Ren Shen), attributed to the strong hydrogen-bonding interactions between DES components and target molecules [31] (the herbal powders untreated or treated by three solvents and FTIR analysis of different solvents before and after extraction shown in Fig. 3A). Moreover, DES-based extractions often require shorter processing time and lower temperature, preserving heat-sensitive compounds that may degrade under traditional extraction conditions [32]. Despite these advantages, challenges remain in optimizing DESs formulations for different herbal matrices and scaling up the process for industrial applications. Future research should focus on standardizing DES-based extraction protocols, evaluating solvent recyclability, and assessing the biological safety of residual DESs in final herbal products [33].

Besides that, DESs enable efficient low-temperature extraction that preserves heat-sensitive bioactive compounds in Chinese medicines, which would otherwise degrade under conventional high-temperature extraction methods. This advantage is particularly valuable for thermolabile constituents such as polysaccharides, whose structural integrity and bioactivity are better maintained through DES-mediated extraction. Similarly, volatile oil components from herbs like *Angelica sinensis* (Danggui) remain stable when extracted using DESs at mild temperatures, preventing the evaporation and decomposition of these delicate aromatic compounds [34]. The ability of DESs to effectively solubilize these compounds at reduced temperatures represents a significant advancement in herbal extraction technology, combining extraction efficiency with compound stability.

As summarized in Table 2, DESs consistently outperform conventional organic solvents in terms of extraction yield, compound selectivity, and environmental safety. Their superior solubilizing capacity is largely attributed to the strong H-bonding interactions and polarity matching between DESs components and target molecules. The efficiency of DES-mediated extraction is influenced by several key parameters, including the specific composition of the DESs, the proportion of water added to modulate viscosity and polarity, the extraction temperature, and the duration of the process. For instance, DESs based on choline chloride combined with glycerol or urea have demonstrated remarkable efficacy in extracting polar compounds while maintaining their structural integrity and bioactivity. These systems not only enhance solubility but also reduce degradation during processing.

**Table 2:** Comparison of DESs for extracting bioactive compounds from some representative Chinese medicines

Herbs	Target compounds	DES composition (HBA: HBD, molar ratio)	Extraction conditions (Temp/Time)	Yield (%)	Reference solvent	Yield with reference (%)	Remarks	Ref
*Scutellaria baicalensis*	Baicalin, Wogonin (Flavonoids)	Choline chloride-Glycerol (1:2)	60°C/45 min	78.5	Ethanol (70%)	65.2	Enhanced solubility and selectivity	[30]
*Coptis chinensis*	Berberine, Palmatine (Alkaloids)	Choline chloride-Urea (1:2)	50 °C/60 min	82.3	Methanol	70.4	Strong hydrogen bonding with alkaloids	[35]
*Panax ginseng*	Ginsenosides Rb1, Rg1 (Saponins)	Choline chloride-Lactic acid (1:2)	70 °C/30 min	74.6	Water	58.9	Preserves bioactivity, low degradation	[31]
*Astragalus membranaceus*	Astragaloside IV, Polysaccharides	Choline chloride-Malic acid (1:1)	80 °C/90 min	69.1	Hot water	52.7	Improved purity and yield	[36]
*Ginkgo biloba*	Quercetin, Kaempferol (Flavonoids)	Choline chloride- Sorbitol (1:2)	55 °C/60 min	75.4	Acetone–water (50%)	62.1	Low toxicity, high antioxidant recovery	[10]
*Rhizoma Corydalis*	Tetrahydropalmatine (Alkaloid)	Choline chloride-Citric acid (1:1)	60 °C/40 min	76.8	Ethanol (60%)	61.3	Enhanced penetration and release	[10]
*Salvia miltiorrhiza*	Tanshinones, Salvianolic acids	Choline chloride- Glycol (1:2)	65 °C/50 min	81.2	Methanol	68.5	Dual polarity DES improves extraction	[37]

Furthermore, DESs can be synergistically integrated with advanced extraction technologies such as ultrasound-assisted extraction (UAE) and microwave-assisted extraction (MAE). UAE can maintain the structure and activity of the extract while accelerating the release, diffusion, and entry of soluble target substances into the extractant, which is time-saving and efficient. DESs can promote the breakdown of hydrogen bonds between raw material skeletons, making the target components more easily soluble and thus improving the extraction rate; these two can work together in synergy. The presence of DESs is beneficial for increasing the movement speed of medium molecules, enhancing the penetration power of the medium, and assisting in the lysis of cell walls and the entire organism by micro shock waves. In addition, the larger heat capacity can also reflect the thermal effect of ultrasound to a greater extent. As for MAE, microwave radiation can realize extremely strong penetration power; in addition to its ability to induce intracellular water evaporation and cell rupture, the strongly polar DES molecules that form the liquid film on the surface of solid materials will also instantly polarize and undergo high-frequency polarity transformation motion. This causes the liquid film attached to the solid phase to be disturbed and become thinner, thereby reducing the resistance of the solid–liquid leaching diffusion process and promoting the diffusion process. In summary, these assisted approaches significantly accelerate mass transfer, reduce solvent consumption, and improve overall efficiency, making them highly suitable for both laboratory-scale and pilot-scale applications. Table 3 provides a series of successful DES-based UAE and MAE research examples.

**Table 3:** DESs-based UAE and MAE for bioactive compounds from representative Chinese medicines

Modes	DES composition (HBA: HBD, molar ratio)	Extraction conditions	Target compounds	Extraction rates	Ref
UAE	1,4-Butanediol-Malonic acid (1:2.5)	Water content in DES: 18%, 1 g: 30 mL, 500 W, 30 min	Phenylethanoid glycosides in *Cistanche deserticola*	1.82 ± 0.01%	[38]
	Choline chloride-Lactic acid (1:4)	Water content in DES: 25%, 1 g: 56 mL, 250 W, 65 min	Saponins in* Abrus cantoniensis Hance*	26.569 mg/g	[39]
	Choline chloride-Lactic acid (1:4)	Water content in DES: 30%, 1 g: 50 mL, 60 W, 10 min, 70 °C	Plantamajoside and acteoside in* Plantago Asiatica L*	8.43 ± 0.58 mg/g 5.93 ± 0.46 mg/g	[40]
	Choline chloride-Lactic acid (1:2)	Water content in DES: 22%, 1 g: 40 mL, 330 W, 18 min	Saikosaponin	16.25 ± 0.42 mg/g	[41]
	Choline chloride-Glycol (1:4)	Water content in DES: 41.7%, 1 g: 52.4 mL, 200 W, 40 min, 62.5 °C	Glycyrrhizin	10.727 mg/g	[42]
	Choline chloride-Glycerol (1:2)	Water content in DES: 26%, 1 g: 38 mL, 100 W, 30 min	Salidroside in *Rhodiola rosea L*	11.64 mg/g	[43]
	Choline chloride-Urea (1:2)	Water content in DES: 45%, 1 g: 15 mL, 300 W, 45 min, 55 °C	Geniposide, crocin I, and crocin II in *Gardenia jasminoides*	48.44, 6.17, 0.81 mg/g	[44]
	Choline chloride-1,4-Butanediol (1:4)	Water content in DES: 30%, 1 g: 30 mL, 200 W, 40 min, 90 °C	Polysaccharides in *Dioscorea opposita* Thunb	15.91%	[45]
	Choline chloride-Urea (1:2)	Water content in DES: 20%, 1 g: 10 mL, 360 W, 30 min, 40 °C	Polysaccharides in* Jujube*	8.33 ± 0.26%	[46]
	Choline chloride-Isopropanol (1:3)	Water content in DES: 40%, 1 g: 50 mL, 250 W, 30 min, 39 °C	Glycyrrhiza polysaccharide	8.31%	[47]
	Choline chloride-Glycerol (1:2)	Water content in DES: 30%, 1 g: 39 mL, 90 W, 30 min, 58 °C	Polysaccharides in *Phellinus igniarius*	13.11 ± 0.16%	[48]
	Choline chloride-1,3-Butanediol (1:2)	Water content in DES: 11%, 1 g: 11 mL, 500 W, 41 min, 70 °C	Polysaccharides in* Moghania macrophylla*	2.47 ± 0.03%	[49]
	Choline chloride-Glycerol (1:3.4)	Water content in DES: 50%, 1 g: 20 mL, 150 W, 10 min, 40 °C	1-deoxynojirimycin in Mulberry Leaves	1.445 mg/g	[50]
	Betaine-Glycerol (1:3)	Water content in DES: 20%, 15 mg: 1 mL, 300 W, 30 min	Esculin, fraxin, aesculetin and fraxetin extr (coumarins) in *Cortex Fraxini*	36.26 mg/g, 30.14 mg/g, 1.87 mg/g, and 9.98 mg/g	[51]
	Choline chloride-Lactic acid (1:2)	Water content in DES: 20%, 1 g: 20 mL, 600 W, 40 min, 45 °C	Flavonoids in *Polygonatum kingianum*	17.13% ± 0.25%	[52]
	Choline chloride-1,4-Butanediol (1:3)	Water content in DES: 28%, 1 g: 25 mL, 450 W, 38 min, 65 °C	Flavonoids in *Chrysanthemum indicum*	62.16 mg/g	[53]
	Choline chloride-Acetic acid (1:2)	Water content in DES: 0%, 0.1 g: 30 mL, 90 W, 82 min, 42 °C	Naringin, hesperidin, and citroside in *Citrus aurantium*	3.36%, 0.629%, 15.96%	[54]
MAE	N_4444_Cl-Decanoic acid (2:1)	Water content in DES: 33%, 0.5 g: 10 mL, 700 W, 16 min, 85 °C	Baicalin in *Scutellaria baicalensis*	66.99 mg/g	[55]
	Choline chloride-Sorbitol (1:2)	Water content in DES: 31%, 1 g: 38 mL, 450 W, 60 min	Flavonoids in raspberry	5.88%	[56]
	Choline chloride-1,2-Propanediol (1:2)	Water content in DES: 40%, 1 g: 50 mL, 600 W, 40 s, 40 °C	Anthocyanins in mulberry	35.97 mg/g	[57]
	Choline chloride-Oxalic acid (1:2)	Water content in DES: 50%, 1 g: 30 mL, 350 W, 11 min, 59 °C	Flavonoids in *Eucommia ulmoides*	3.09%	[58]
	Choline chloride-Glycerol (1:2)	Water content in DES: 20%, 1 g: 20 mL, 600 W, 18 min, 66 °C	Phenols in mulberry leaves	8.352 mg/g	[59]
	Choline chloride-Acetylpropionic acid (1:2)	Water content in DES: 30%, 1 g: 15 mL, 160 W, 75 min, 90 °C	Lignanoids in magnolia bark	32.87 mg/g	[60]
	Choline chloride-Citric acid (1:1)	Water content in DES: 20%, 0.012 g: 1 mL, 80 W,16.46 min	Aloe-emodin, emodin, chrysophanol, and physcion in *Rheum palmatum*	2.29, 2.32, 35.44, and 20.80 mg/g	[61]
	Choline chloride-1,2-Propanediol (1:3)	Water content in DES: 20%, 1 g: 25 mL, 1000 W, 330 s, 95 °C	Forsythoside A in *Forsythia suspensa*	137.62 mg/g	[62]
	Choline chloride-Ethylene glycol (1:6)	Water content in DES: 20%, 1 g: 60 mL, 800 W,90 min, 100 °C	Cinnamaldehyde in *Cinnamomum cassia* twigs	12.64 mg/g	[63]

#### Limitations and considerations in DES-based extractions

Despite their many advantages, it is important to recognize that DESs are not universally superior to conventional solvents in all extraction contexts. In some cases, the strong hydrogen-bonding network between the HBA and HBD can dominate the solvent structure, reducing its availability to interact with target solutes. This can lead to lower extraction efficiency, especially for compounds that do not participate readily in the DES’s hydrogen-bonding framework or that have steric or polarity mismatches with the DES phase. For instance, when the DES components are highly self-associated, the solvent may exhibit poor solvation capacity for certain non-polar or sterically hindered bioactive molecules. Such limitations highlight the importance of rational DES design and preliminary screening when selecting a solvent for a specific herbal matrix or target compound. Therefore, while DESs offer a versatile and green alternative, their performance must be evaluated on a case-by-case basis, considering factors such as solute–solvent affinity, viscosity, and phase behavior.

#### Physically‑assisted DES extraction methods

Since DES-based MAE and UAE both have their own characteristics and advantages, combining the two is a new method worth exploring. Yu and coworkers weighed 1.5 g of dried powders of mulberry fruit and then added the extractant of DESs with a certain water content (20–60%) in a solid–liquid ratio of 1:5–1:25 g/mL; furthermore, ultrasonic extraction (180–300 W) followed with microwave extraction (300–600 W) under different temperature, power, and time were investigation. As the result, when using choline chloride-lactic acid (1:1) as DESs, with a water content of 50%, a solid–liquid ratio of 1:15 g/mL, ultrasound temperature of 60 °C, ultrasound power of 300 W, radiation time of 30 min, microwave power of 750 W, and radiation time of 40 s, the optimal extraction rate of anthocyanins is 8.892 mg/g, and its antioxidant capacity is stronger than that of ascorbic acid [64]. However, the researchers did not explore the sequence of the two auxiliary extraction modes and compare the effectiveness with using them separately to explore the synergistic effect.

Overall, due to the late emergence of DESs compared to ILs, the former is more active in current innovation of extraction methods, and some new strategies and devices have also emerged for various purposes. In theory, most of the new technologies suitable for ILs are also applicable to DESs, so they can inspire and learn from each other. In addition, there are also some unique methods that specifically utilize the characteristics of DESs. Making the target object form DES in situ is a good idea. For example, Cao et al. [65] mixed the powders of *Dendrobium devonianum* Paxton with L-carnitine as HBA in water, with a solid–liquid ratio of 0.16 g/mL for the former and a concentration of 0.003 mol/mL for the latter in the system. As designed, the extracted polysaccharides formed DESs with L-carnitine and were fully dissolved under the effect of cell disruptor, with a final extraction rate of 79.3%. Compared with the result of extracting directly with water without adding L-carnitine (34.0%), there is a significant improvement, and the performance was better and the time was shorter than using heating, ultrasound, or microwave-assisted extraction. Moreover, the DESs systems with phase switchable properties induced by pH change or introduction of CO_2_ also has incomparable advantages in extraction and recovery [66, 67].

**Fig. 4:**
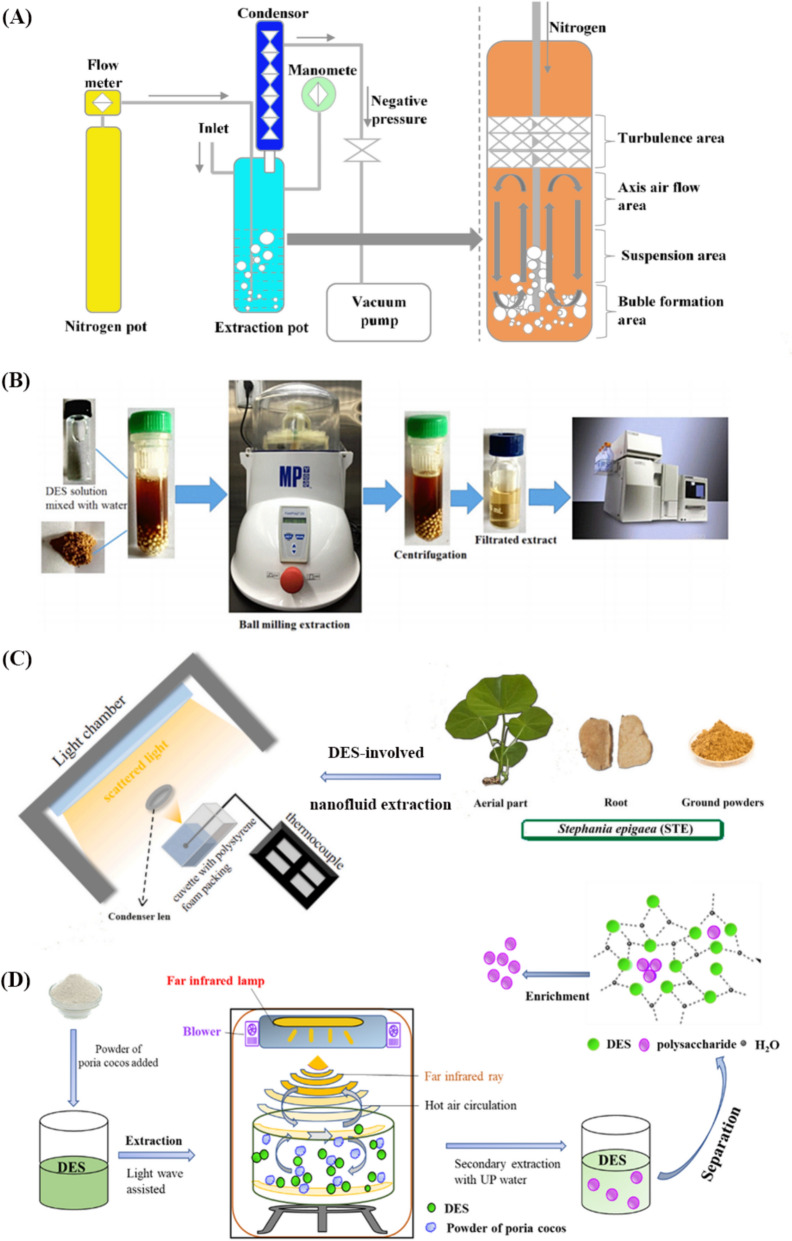
**A** Typical NPCE instrument, **B** ball-mill extraction [71], **C** nanofluid using zirconium carbide and DESs with photo-assisted extraction [72] and **D** DES-FIR-HACE scheme

In order to achieve high extraction efficiency, pressurized and negative pressure devices are two completely different choices. Of course, the operational risk of the former will be greater. The choline chloride-lactate (1:1) system has been used to extract the main components of compound Chinese medicine under ultra-high pressure (100*–*600 MPa) conditions [68]. The powders of compound Shuanghuanglian preparation composed of honeysuckle flower, *Scutellaria baicalensis*, and *Forsythia suspensa* were packed together with the DES (water content: 40%) in a polyethylene bag and vacuum sealed; then it was put into the ultra-high-pressure equipment, which corresponded to a set of HLM-100-300S high-pressure processing machines (Shanshui Yinhe Sci & Tech., Co. Ltd., Taiyuan, China) for the food industry. In the initial stage, an increase in pressure has a positive correlation with extraction efficiency, which helps the solvent diffuse into the herbal cells and reduces the mass transfer resistance of active ingredients by breaking cell wall. When the pressure exceeds 400 MPa, the higher pressure increases the degree of fragmentation of the raw materials, forming too many small particles that block the dissolution channels of the active ingredients. At the same time, some of them can be adsorbed by the fragmented cells, resulting in a decrease in extraction efficiency. It can be found that plant cells rupture rapidly under 400 MPa within 4 min, and the extraction efficiency has reached its maximum. During the pressure relief process, the sudden decrease in pressure allows the active ingredients to pass through multi-layers of cells and transfer to the extracellular space, thus also promoting efficient extraction. Differently, negative pressure cavitation is the process of creating a negative pressure zone in a liquid medium through physical action, resulting in the formation of cavitation bubbles. As the bubbles gradually expand and frequently collapse, they generate enormous pressure and repeatedly impact the surrounding environment. Negative pressure cavitation extraction (NPCE, see Fig. 4A) technology utilizes the mechanical and thermal effects associated with cavitation. The former is manifested in the enlargement of heterogeneous reaction interfaces, rapid rupture of plant cell walls, and accelerated release, diffusion, and dissolution of intracellular substances into the medium; the latter utilizes the high temperature and pressure generated during the cavitation effect to accelerate the extraction process of target components, such as molecular decomposition and chemical bond breaking. Negative pressure is a key factor in improving DESs extraction efficiency [69]. Within a certain negative pressure value, the lower the negative pressure, the more intense the cavitation effect, which promotes the mixing of raw materials and DESs and then strengthens mass transfer. However, excessively low negative pressure actually reduces air flow, and if there is not enough air to form a stirring effect, it will affect mass transfer. In addition, increasing the temperature can help reduce the viscosity of DESs and enhance diffusion, but prolonged high temperature can lead to the decomposition of thermally unstable targets, so researchers need to optimize the conditions to avoid these problems. With the rapid development and emergence of the discipline of mechanochemistry, ball-milling is a meaningful method that utilizes the kinetic energy provided by high-speed rotation to grind and crush raw materials, achieving the effect of destroying their fiber skeleton and extracting target natural components [70]. Based on this principle, it can quickly and effectively extract natural products from raw cells. Wang et al. developed a ball-mill assisted DESs extraction technology to replace traditional reagents used to extract tanshinone from *Salvia miltiorrhiza* (see Fig. 4B) [71]. Combining ball-milling with DESs has the advantages of green and high efficiency. For solid–liquid extraction, the solubility of DESs largely determines the extraction performance, which is related to its diffusion, solubility, viscosity, surface tension, polarity, and physicochemical interactions during the process. These factors often constrain each other during the extraction process, such as stronger electrostatic forces and hydrogen bonding interactions with the targets. Therefore, higher viscosity and lower molecular migration rate lead to a decrease in the permeability of the target molecule, while a decrease in surface tension can effectively improve the permeability of the extractant. In addition, an interesting phenomenon can be observed through scanning electron microscopy, that is, the morphology of herbal powders extracted by different solvents varies greatly when using the same method. After ball-milling extraction with methanol, the raw material will become particles, while after extraction with DESs solution, the particles can “melt” and form porous sheets. This phenomenon is caused by the destruction of the cellulose skeleton in herbal particles by DESs, which reflects its unique and advantageous aspect for extraction.

Finally, some technological means that have been widely applied in fields such as new energy in recent years can also be used as a reference for the extraction field, and interdisciplinary collaboration provides a continuous driving force for continuous innovation. As a potential powerful natural lead compound against COVID-19, cepharanthine (CEP) has attracted much attention during the global epidemic. Our group successfully prepared a nanofluid using zirconium carbide and DESs with photothermal conversion properties, which was used for the photocatalytic extraction of CEP from *Stephania epigaea* (see Fig. 4C). The final yield of CEP was approximately four times higher than that of heating method with ethanol, and the Fick’s model was well applied to fit the kinetic data. Compared to traditional extraction methods for alkaloids, the newly developed method is more efficient, friendly, and energy-saving [72]. Besides that, we attempted to irradiate a mixture of herbal powders and DESs with far-infrared light (100*–*500 W/m^2^) to accelerate the movement of molecules. At the same time, the use of vortex airflow evenly distributed around the mixture at 360 °C makes heating more uniform, and the water generated during the extraction process can be immediately taken away. In addition, the surface and interior of the herbal powders are fully penetrated by the hot air flow, causing the raw material to burst and become very easy to be penetrated by DESs. Through the so-called far infrared radiation-hot air circulation-assisted deep eutectic solvent-based extraction (DES-FIR-HACE, see Fig. 4D), the target component can rapidly diffuse from the cells into the extractant. Under such mild conditions, some thermally unstable substances will not be affected by excessively high temperatures. Therefore, this new process has the advantages of low time consumption, high extraction efficiency, environmental friendliness, and maintaining the bioactivities of ingredients [73].

### Advanced separation strategies using DESs

Besides the applications for crude extraction, DESs have shown great potential in LLE and ABS for the selective separation of bioactive compounds from Chinese medicinal extracts. Unlike conventional organic solvents, DESs can form immiscible phases with water or other solvents, enabling efficient partitioning of target molecules based on their polarity and solubility.

#### Liquid–liquid separation systems

Due to a later start, the number of existing LLE literature of DESs is relatively smaller than that of ILs. On the one hand, the continuously accumulated phase equilibrium data of green solvent systems can provide strong support for method development. On the other hand, the COSMO-RS model can also be used to reduce experimental workload in screening of DES-involved systems, and the high extraction performance of the screened DESs can be analyzed by combining the shielding conductor charge density distribution (σ-profile) and chemical potential curve (σ-potential). Such a kind of green solvent is particularly safe and friendly when processing drugs, health products, foods, cosmetics, and other objects in liquid–liquid extraction mode. Similarly, its structural composition and the main operational conditions also directly affect the extraction efficiency and selectivity of various natural bioactive constituents in TCM, including extraction temperature, liquid–solid ratio, extraction time, and sample concentration. DES-enhanced LLE enables efficient and selective separation of bioactive compounds from Chinese herbal extracts, where hydrophobic DESs (e.g., thymol-menthol) effectively extract non-polar constituents (terpenoids, coumarins) from aqueous phases, while hydrophilic DESs (e.g., choline chloride-urea) preferentially isolate polar molecules such as flavonoids and phenolic acids. This approach demonstrates superior performance over conventional methods, achieving > 90% recovery of alkaloids like berberine from *Coptis chinensis*—significantly outperforming traditional ethyl acetate partitioning [74]. Furthermore, DES-water systems exhibit markedly reduced emulsification compared to organic solvent–water mixtures, facilitating cleaner phase separation and substantially simplifying downstream processing. As for how to apply related process and conditions in actual industrial production, the article titled as “Scaling-up liquid–liquid extraction experiments with DESs” introduced a meaningful example of DES liquid–liquid extraction research from 10 to 1000 g [75], and interested readers can gain a deeper understanding of the relevant strategy.

With the introduction of the principle of GAC, some researchers further miniaturized the extraction solvent on the basis of traditional liquid–liquid extraction for further quantitation, in order to improve the sensitivity of analysis and extraction efficiency [76]. As an HBA, alkyl quaternary ammonium salt can form stable and well-flowing DESs with long-chain fatty acids or alcohols. According to the principle of 'similar compatibility’, it can be used to extract non-polar active ingredients in Chinese herbal medicines. The DESs prepared by tetrabutylammonium chloride (TBAC) and hexanoic acid (HA) at a molar ratio of 1:2 at 100 °C  was artificially shaken for 15 s under the optimal two-phase conditions. The average enrichment values of four natural bioactive components of caffeic acid, p-hydroxycinnamic acid, ferulic acid, and cinnamic acid in the methanol solution of *Angelica sinensis* and *Ligusticum sinense* ‘Chuanxiong’ could reach 135–220 [77]. In the process of artificial shaking, DESs and sample solution were quickly dispersed into tiny droplets, which greatly improved the contact area between the two phases, so that the target compound could achieve rapid and sufficient mass transfer between the two phases, greatly shortened the extraction time and improved the extraction efficiency to a certain extent. It is a simple, efficient, economical and green extraction method. *Codonopsis pilosula* (CP) is a perennial herbaceous vine mainly distributed in northern China. According to the different producing areas, it can be roughly divided into Lu Dangshen and Wen Dangshen. Since the Qing Dynasty, it has been used as a traditional Chinese herbal medicine. It has the effects of generating blood, protecting the intestines and stomach, and enhancing immunity, and it can also be used as nourishment to add to daily diet. The experimental personnel sieved the CP powder and added it into the methanol solution. After ultrasonication and filtration, the sample solution was obtained. Four DESs based on methyltrioctylammonium chloride (TOMAC) were added to it for extraction. Then the mixed solution was centrifuged and the supernatant was diluted with methanol for HPLC analysis [78]. The experimental results showed that when the pH of the sample solution was 7, 70 μL of TOMAC-glycerol with a molar ratio of 1:4 was added as an extractant, the best extraction effect could be achieved by centrifugation for 2 min after vortexing for 90 s. The enrichment factors of the five small molecule active components of lobetyolin I, lobetyolin, lobetyolin, lobetynol and atractylenolide III in CP were 6.0, 6.2, 18.9, 58.7 and 63.2, respectively, which confirmed the superiority of TOMAC-glycerol extraction.

#### ABS with DESs

**Fig. 5:**
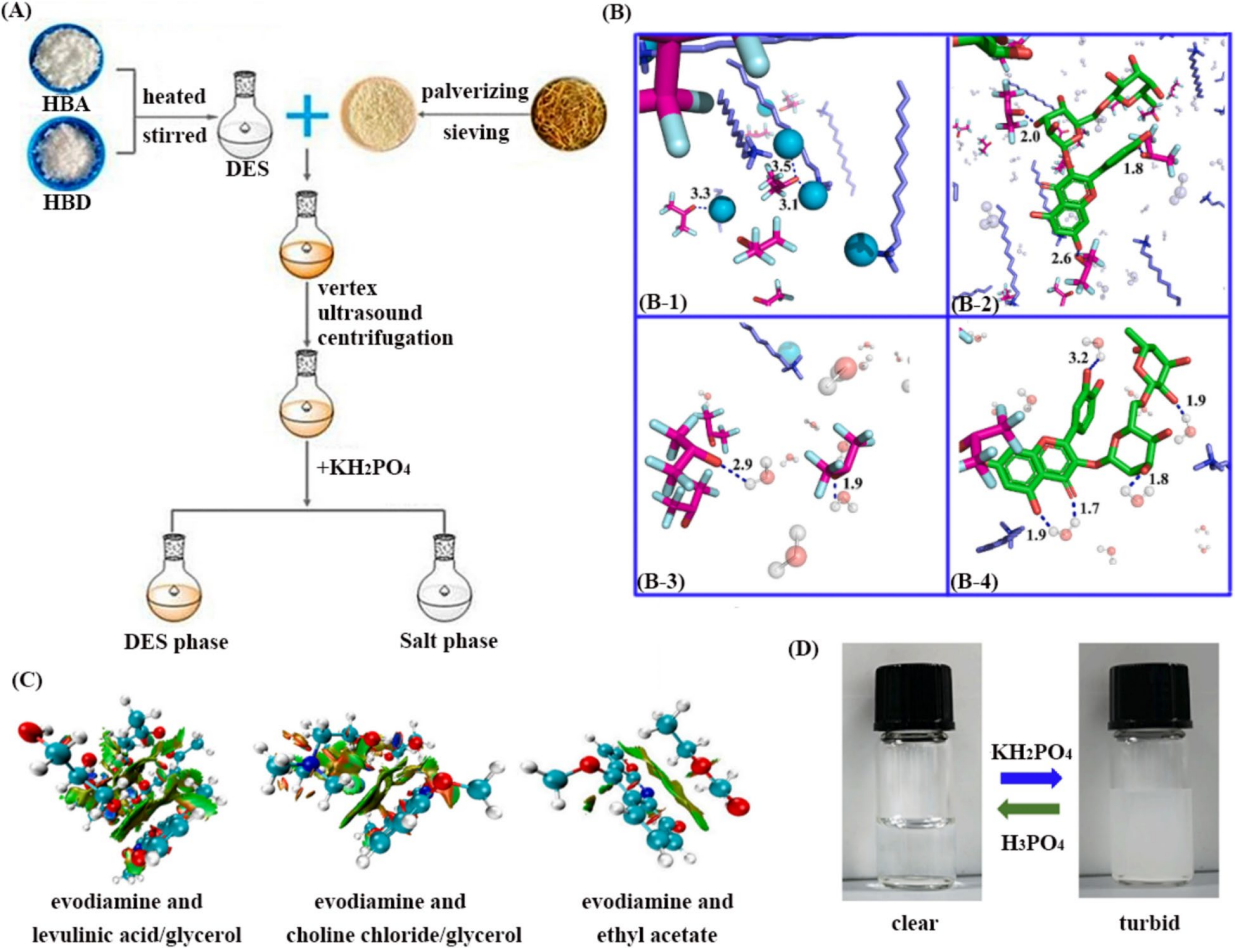
**A** DES-ABS for for sustainable recovery of ginsenosides from *Panax quinquefolius* L. [80]; **B** bonding interactions between **B**-**1** HBA and HBD of the DES, **B**-**2** DESs and rutin, **B**-**3** water and DES, **B**-**4** water and rutin [81]; **C** interactions between evodiamine and various solvents [82] and **D** a pH-responsive DES-ABS [83]

ABS incorporating DESs offer a mild and energy-efficient approach for fractionating heat-sensitive compounds from Chinese herbal extracts. These systems, typically composed of DESs with salts or polymers, enable selective separation based on compound solubility and partitioning behavior. For polysaccharide purification, DES-ABS (e.g., choline citrate/K_2_HPO_4_) effectively precipitate Astragalus polysaccharides (AMP) while retaining small-molecule impurities in the aqueous phase [79]. In details, 0.2 g of dried Astragalus powders and a certain volume of (choline chloride:urea = 1:1) were mixed in a 10 ml centrifuge tube, and then AMP was extracted by ultrasonic-assisted heating. After centrifugation, a certain amount of K_2_HPO_4_ solution were added in the the supernatant (the crude polysaccharides extract), and the mixture was fully stirred. After centrifugation again, two phases were formed and AMP was enriched in the bottom layer. As the result, the optimal extraction efficiency was obtained as 97.85%. Similar to polysaccharides, there are also large polar components in TCM such as saponins. The biphasic systems commonly used for separating saponins include: polymer–polymer systems (polyethylene glycol ((PEG))-Dextran), polymer-salt systems (PEG-sulfate, phosphate, or citrate), alcohol-inorganic salt systems (ethanol, isopropanol, or propanol-(NH_4_)_2_SO_4_), IL-salt systems ([C_4_mim] Cl, or [C_4_mim]BF_4_-phosphate or citrate), etc. The specific choice of system needs to be comprehensively considered based on the properties of the target saponins, production costs, environmental requirements, and the overall connection between upstream and downstream processes. In order to remove the polysaccharides coexisting in the crude saponins extract, Wang and his colleagues found the optimal aqueous biphasic system composed of 80% (w/w) choline chloride-N,N-dimethylurea and 60% (w/w) K_2_HPO_4_ (see Fig. 5A), achieving a remarkable ginsenoside yield of 73.20 mg/g (54.6% higher than existing systems) from *Panax quinquefolius L* within 30 min [80]. The mechanism of ginsenoside extraction using the DESs was revealed by quantum chemical (QC) calculations combined with molecular dynamics (MD) simulations; related results proved the hydroxyl group in ginsenosides could simultaneously act as both HBA and HBD, promoting interactions between Cl^−^ and HBD within the DESs. The comparison for above four types of ABS is included in Table 4.

**Table 4:** Comprehensive comparison on four typical ABS

No	Types	Examples	Merits	Demerits	Applicable scenarios
1	Polymer–polymer system	PEG-Dextran	Mild phase separation, high resolution,	High cost, high viscosity	Laboratory fine separation, basic research
2	Polymer-salt systems	PEG-K_2_HPO_4_	Low cost, easy to scale up, fast phase separation	High salt wastewater, requiring post-treatment	Preferred for industrial large-scale production
3	Alcohol-inorganic salt system	Ethanol-(NH_4_)_2_SO_4_	Low cost, good connection with extraction process, high recovery rate	Alcohol loss for the volatilization	Great application prospects, especially suitable for ethanol extract
4	IL/DES-salt system	[C_4_mim]Cl-K_2_HPO_4_, choline chloride-urea- K_2_HPO_4_	Green, high selectivity, and ideal designability	High cost if the system cannot be reused	Laboratory research on the separation of high-value products

#### Water-mediated structural changes and partitioning behavior in DES-ABS

It is important to recognize that in many DES-based ABS, the introduction of water whether as part of the phase-forming components or as a modifier can significantly alter the solvent microstructure. Excess water molecules can compete with and disrupt the hydrogen-bonding networks between the HBA and HBD, leading to partial dissociation of the DESs. This phenomenon often results in differential partitioning of the HBA and HBD between the two aqueous-rich phases, which in turn influences the distribution of target bioactive compounds. For instance, in systems where DESs components exhibit contrasting hydrophilicity (e.g., choline chloride as HBA and a carboxylic acid as HBD), water addition can shift the phase equilibrium and modify the polarity and solvation properties of each phase. Consequently, the extraction selectivity and efficiency in DES-ABS are not only determined by the initial DES composition but are also dynamically governed by water content and its interaction with the DESs components. This behavior underscores the need to carefully optimize water content and system composition when designing DES-ABS for the separation of specific TCM constituents.

Flavonoids such as rutin and quercetin, which are beneficial to human body, have high content in *Flos Sophorae* Immaturu and exhibit pharmacological effects such as anti-inflammatory, anti-oxidation, heat-clearing and blood-cooling. Yu et al. constructed ABS with DESs made of dodecyl trimethyl ammonium chloride (DTAC) and hexafluoroisopropanol (HFIP) at a molar ratio of 1:2 and K_2_HPO_4_, then used it for the extraction of flavonoids in FSI, which could avoid the degradation of flavonoids caused by high temperature in traditional extraction methods. Under the optimal conditions, the yield of flavonoids was 21.05% [81]. In this system, DTAC has a long alkyl chain, which can effectively improve the hydrophobicity of DESs to promote the effective enrichment of flavonoids. As a salting-out agent, K_2_HPO_4_ can not only promote the dissolution of flavonoids from plant cells, but also promote the increase of hydrogen bond network and π-π stacking between rutin molecules (see Fig. 5B), which can further improve the extraction efficiency. Sometimes, the target components are not all enriched in the DES phase and can also be captured by another artificially introduced low polarity phase, depending entirely on the researcher’s design. For example, after extracted with the DESs of levulinic acid–glycerol with molar ratio of 1.5:1 containing 30% K_2_HPO_4_ solution, the bioactive alkaloids in the residue of the Chinese herb *Evodia lepta* could be further enriched by ethyl acetate phase. The total alkaloids with highest yield of 757.71 μg/g, 587.45% higher than that achieved using traditional methanol extraction; they were transferred to the ethyl acetate phase with selectivity of 72.03, 39.16, and 80.67% for skimmianine, dictamnine, and evodiamine, respectively [82]. The DFT study proved that the present DESs system possesses higher capacity of forming H-bonding with alkaloids than other tested solvents (see Fig. 5C), and only weak interactions of H-bonds and van der Waals forces formed during the extraction process rather than strong covalent bonds. Furthermore, there are no obvious changes occurring within the DESs configuration, indicating the recyclability and stability of the green solvents. In a sense, the above strategy is equivalent to incorporating the post treatment process of back-extraction into the extraction process to complete all the tasks in one step; here it is suggested to replace volatile ester solvents with green solvents (e.g., γ-valerolactone, propylene carbonate, etc.) of the same polarity to achieve overall greenness.

Similar to the strategy in previous section about the innovations in extraction, various responsive systems have provided new options for researchers. For instance, pH-responsive DES-ABS (see Fig. 5D) facilitates alkaloid enrichment through pH-dependent partitioning, as demonstrated by the efficient extraction of aromatic amino acids [83], as well as ephedrine from *Ephedra sinica*; and this strategy also achieved the combination of ILs and DESs in extraction and separation. In summary, above DES-ABS techniques provide significant advantages over conventional methods, including lower energy requirements, enhanced selectivity, and improved preservation of thermolabile phytochemicals. While DES-ABS show great promise for herbal extraction, several critical challenges must be addressed for widespread adoption. The efficient recovery and recycling of DESs from ABS remains technically challenging due to their complex interactions with target compounds and phase-forming components. Due to the relatively complex composition as well as the fact that the optimal phase-forming conditions may not necessarily be the optimal extraction conditions, these poses significant challenges for establishing the entire system. To optimize system design, researchers are developing ML models capable of predicting DES-salt/polymer phase behavior, which could significantly accelerate the screening of optimal extraction conditions [84]. Addressing these challenges through continued research and industry collaboration will be essential for realizing the full potential of DESs-based extraction technologies in R&D of TCM.

#### HSCCC with DESs

HSCCC is a continuous and efficient liquid–liquid distribution chromatography separation technique developed in the 1980s. It does not require any solid support or carrier, and both the stationary and mobile phases are liquid. HSCCC utilizes a two-phase solvent system to establish a special unidirectional fluid dynamic equilibrium within a high-speed rotating helical tube, with one phase serving as the stationary phase and the other as the mobile phase. During separation process, the stationary phase is distributed in a relatively uniform manner in a helical tube, and the directionality and synchronous planetary motion of the helical tube generate a two-dimensional centrifugal force field, forming a unidirectional fluid dynamic equilibrium. When the mobile phase moves at a certain speed, the stationary phase is retained, and the separated substances are sequentially eluted due to their different distribution coefficients, resulting in separation. Due to its advantages of no sample loss, strong loading capacity, large preparation quantity, and good reproducibility, HSCCC has been widely used for the separation of TCM components (including alkaloids, flavonoids, polyphenols, anthraquinones, coumarins, terpenes, proteins, etc.) [85]. The solvent system is the most key factor affecting the separation efficiency of HSCCC, and it needs to be systematically explored before the start of separation work. The three classic systems include chloroform methanol water (4:3:2, V/V), n-hexane methanol water (1:1:1, V/V), and n-hexane ethyl acetate methanol water (1:1:1:1, V/V) [86]. Generally, the organic phase with low density (upper phase) is used as the stationary phase, while the aqueous phase with high density (lower phase) works as the mobile phase. The specific choice of solvent system for the separation of the target substance usually depends on whether the distribution coefficient of the substance in the solvent system is within an appropriate range. The distribution coefficient (K) can be determined by shaking the bottle method, and its specific value is equal to CS/CM, where CS refers to the concentration of solute in the stationary phase and CM refers to the concentration in the mobile phase. The usual suitable range of K is 0.5–2. When CS/CM ≤ 0.5, the peak time is too fast and the separation between peaks is poor; When CS/CM ≥ 1, the peak time is too long and the peak deformation is wide. When 0.5 < CS/CM < 2, a peak shape with good separation can be obtained within an appropriate time. Generally speaking, for the separation of highly polar substances such as organic acids and bases, n-butanol water is commonly used as the basic solvent system for high-speed counter current chromatography. However, this system has a low retention rate of the stationary phase in the counter current chromatography column and is prone to loss, resulting in poor separation efficiency [87]. Due to the unparalleled advantages of using green solvents for the separation of natural products, as well as their low toxicity, non-volatile nature, good stability, wide solubility range, and strong selectivity, DESs as the solvent system for HSCCC is superior to conventional organic solvents. At the same time, the crucial physical properties such as density, surface tension, and viscosity discussed in the second part of this review will affect the stability, stationary phase retention, and separation efficiency of the DES-involved HSCCC system.

**Fig. 6:**
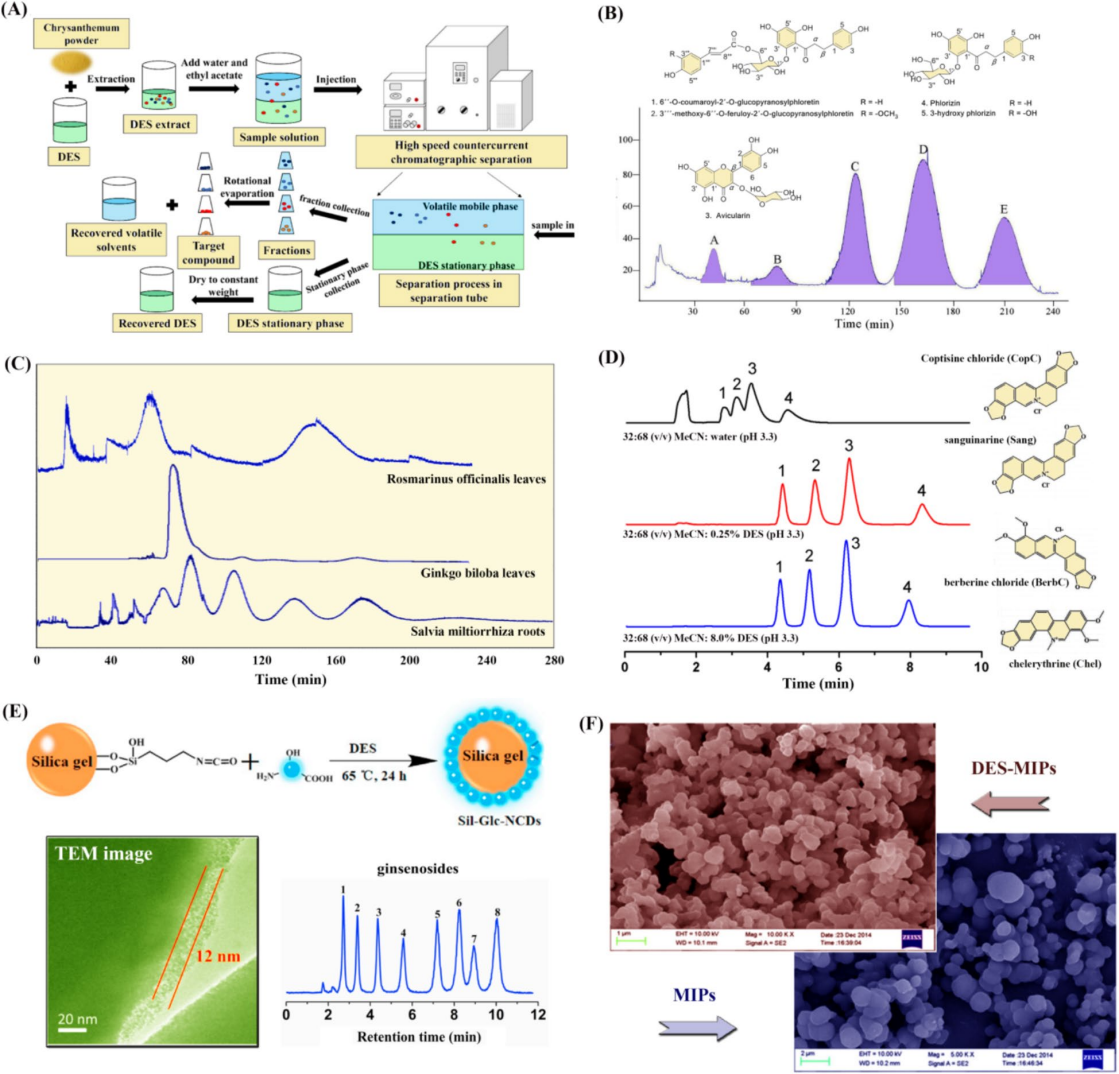
**A** Details of the DESs-involved HSCCC method [88]; **B** HSCCC chromatogram of five flavonoids from Malus hupehensis with choline chloride/glucose-water-ethyl acetate (1:1:2, V/V) [89]; **C** DES-involved HSCCC chromatograms of three herbs [90]; **D** separation of four quaternary alkaloids with different mobile phases [91]; **E** DESs used in the modification of silica gel and the transmission electron microscope of product together with its separation results of gensenosides [93]; **F** comparison on scanning electron microscopic images of DES-MIPs and MIPs [97]

As one of the commonly used TCM, *Chrysanthemum* is the dried inflorescence of *Asteraceae* plants, mainly produced in Zhejiang, Anhui, Henan and other places. Its flowers are harvested in batches from September to November when they are in full bloom, used after drying in the shade or baking. Researchers first prepared a series of DES-based solvent systems and screened them based on their physical and chemical properties [88]. It was found that only the system composed of choline chloride/L-malic acid, water, and ethyl acetate (1:1:1, V/V) was suitable. By using this system for HSCCC separation, the target compounds were successfully separated with high recovery yields over 95%. After HSCCC separation, the solvents in the column were blown out with nitrogen, and the lower DESs phase was collected for reuse after drying (the whole process as shown in Fig. 6A). The results indicated that the proposed method was feasible and efficient, which has great potential for application. Cai and coworkers used DESs for separating high-purity flavonoids from DESs (choline chloride-lactic acid) extract of *Malus hupehensis* [89]. Under the optimal DESs extraction conditions (liquid–solid ratio of 26.3 mL/g, water content of 25.5%, extraction temperature of 77.5 °C), the yield of flavonoids reached 15.3 ± 0.1%, which was superior to the methanol extraction method. The HSCCC solvent system consisted of choline chloride-glucose, water and ethyl acetate (ChCl/Glu-H_2_O-EA, 1:1:2, V/V), resulting in the successful preparation of two new flavonoids and three known flavonoid compounds (1.1–51.1 mg obtained from 100 mg extract, see Fig. 6B). It should be emphasized that ethyl acetate, which is commonly present in the HSCCC system, can dissolve some HBD applied in DESs (such as urea, carboxylic acids, and polyols), so attention should be paid during the development of the separation system. The HBD of glucose used in their study does not have this problem. In addition, the K value of the target flavonoid increases with the increase of ChCl/Glu molar ratio and water content, which is 0.62–1.36 under the volume ratio of 1:1:2 for three components. After separation, the stationary phase containing DESs (with a retention rate of 78%) can be used to further prepare a new solvent system after removing volatile components for recycling.

In addition to the two hydrophilic DESs mentioned above, hydrophobic DESs can also be used for HSCCC. For instance, D,L-Menthol-D,L-Lactic acid (Men/Lac, 1:2) was selected and used to prepare HSCCC solvent systems with other traditional solvents, resulting in DES-petroleum ether-ethyl acetate–methanol-H_2_O (1:3:1:3:1, V/V) and DES-n-hexane ethyl acetate-methnol-H_2_O (1:1:1:1:1, V/V) [90]. They could separate the extracts of *Salvia miltiorrhiza* roots, *Ginkgo biloba* leaves and *Rosmarinus officinalis* leaves into 3–6 fractions (see Fig. 6C). The recovery rate of the main active ingredients in these fractions is all above 90%, and stationary phase retention rates were 73.2%, 71.6% and 69.1% respectively, which were higher than 50% and met the requirements of HSCCC separation. The results proved hydrophobic DESs could be used as HSCCC solvents to separate and recover hydrophobic compounds effectively. It is not difficult to see from the above successful cases that as long as DESs is scientifically selected and evaluated, the HSCCC separation system constructed from it can effectively achieve large-scale preparation of target constituents in TCM, and the applied DESs can also be recycled. At the same time, the types of separated components need to be expanded, and the use of volatile organic solvents has not been avoided in above systems, which undoubtedly affects the overall greenness of the process.

### Solid‑assisted separation systems

#### DES‑based chromatographic stationary phases

Different from above liquid–liquid system, DESs can not only be applied as elution reagents of solid phase or liquid phase, but also be used as raw materials or adjuvants (solvent, dispersant, or pore forming agent, etc.) for preparation of solid phase in liquid–solid systems of this section. Here the first scenario refers to that, when the solid phase binds to the target molecules in TCM through adsorption, affinity, complexation, inclusion, supramolecular effects and so on, the methanol solution of choline chloride-glycerol or choline chloride-urea DESs with a molar ratio of 1:2 can be used as the subsequent eluent, and the recovery rate of related constituents is generally higher than that of ordinary solvents. For the second scenario, DESs can be used in common mobile phase for reverse-phase system (e.g., C_8_ and C_18_ chromatographic columns), and ethanol or acetonitrile are often used in combination of DESs. In addition to forming common reverse phase systems, such combinations can also form hydrophilic chromatographic modes under anhydrous conditions; the DES acts as a strong solvent and its proportion are inversely proportional to the capacity factor of the separated components from TCM. The larger the DESs proportion, the shorter the retention time. Differently, methanol or acetonitrile often play the role of strong solvent in the reverse phase system.

The most concerned issue people often have is whether DESs will affect the lifespan of the expensive chromatographic columns, followed by whether it will improve separation. Qiu et al. used a conventional reverse phase silica gel column (150 mm × 4.6 mm, 3 μm) and a mixed solution of acetonitrile and seven kinds of DESs (choline chloride-ethylene glycol, 1:3; Tetramethyl ammonium chloride-ethylene glycol, 1:3; tetraethyl ammonium chloride-ethylene glycol, 1:3; tetra butyl ammonium chloride-ethylene glycol, 1:3; choline chloride-urea, 1:2; choline chloride-citric acid, 1:1; choline chloride-glycerol, 1:2) as the mobile phase to investigate the chromatographic separation efficiency of four quaternary alkaloids (coptisine chloride, sanguinarine, berberine chloride and chelerythrine) from Chinese medicines (see Fig. 6D). The pH of mobile phase remained at 3.3 and the volume ratio of acetonitrile to DESs aqueous solution was set at 32:68. As the results, both separation and selectivity were improved and the columns were not damaged by using DESs. In detail, choline cations as a part of HBA played a major role on the separation performance, interacting with deprotonated silanols on the surface of silica-based stationary phase, or hindering the access of quaternary alkaloids through adsorption to the C_18_ chains. On the other hand, ethylene glycol as a HBD successfully reduced the retention time. There exist some interactions between DESs and free silanol groups on the surface of the silica, which are reversible and the column is regenerable [91]. As mentioned above, DESs also can be used as raw materials for preparation of solid phase in liquid–solid systems. In fact, the immobilized DESs mentioned in the second section are one of forms to become solid phase. In theory, DESs has the potential to be used for preparing chromatographic stationary phases, but such reports are currently rare. A representative strategy is to use a polymerizable DESs composed of choline chloride and itaconic acid/acrylic acid as functional monomers, and prepare a monolithic column in a polydopamine functionalized polyetheretherketone (PEEK) tube. In addition, DESs has been successfully used for rapid surface modification of spherical porous silica to prepare stationary phases for high-performance liquid chromatography [92]. Compared with organic solvents, the new reaction medium had the advantages of high dispersibility and non-volatility of silicon spheres. This modified stationary phase could be used to separate components such as ginsenosides (see Fig. 6E), flavones, nucleosides and so on, which were often contained in the samples of TCM [93]. When preparing DESs based chromatographic stationary phases using silica gel as a carrier, two main methods can be used: simple coating and chemical method. The latter, such as the substitution reaction between silanol groups and choline chloride, immobilizes DESs composed of the latter and urea (1:2) on normal phase silica. When used to separate ferulic acid, a typical natural organic acid (negatively charged) commonly found in herbs, the chromatographic behaviour exhibited is clearly based on the anion exchange effect occurring on the cation of choline chloride [94]. As for the coating method, due to its high viscosity and ability to form strong hydrogen bonds with silanol hydroxyl groups, DESs is relatively easy to form a fixed phase layer (film) on the surface of silica gel. However, the stability of this hydrogen bond will be affected under the washing of aqueous samples or mobile phases. Possible solutions include: allowing polymerizable HBD and HBA to undergo copolymerization on a carrier, or using hydrophobic DESs as a stationary phase combined with water as eluent, or using hydrophilic DES as stationary phase in normal phase chromatography. The general separation mechanism is mainly based on hydrogen bonding and ion exchange. If Chinese medicine technicians lack experience in preparing DES-involved column chromatographic stationary phases, it is highly recommended to start with the simplest coating method for preparing relevant DES-involved stationary phases for TLC.

#### DES‑modified SPE materials

Besides the applications of DESs in chromatographic stationary phase, they have achieved success in preparation of SPE materials. From the perspective of both preparation ways and separation mechanisms, SPE materials and chromatographic stationary phases have obvious similarities, so they can learn from each other well. Compared to liquid–liquid extraction, SPE technology has made significant progress, which can improve the recovery rate of analytes and resolution between them and interfering components; it reduces sample pretreatment processes, showing the merits of simple operation, time-saving, and labour-saving. The simplest way for TCM technicians to obtain the corresponding SPE medium is to thoroughly mix DES with common carriers such as silica gel and (neutral) alumina, and then wash away the unstably bound DES. For example, after dried in the oven for 3 h, 2 g silica gel (particle size: 5–15 μm) was sonically dispersed into a mixture of 8 mL of the isochoric methanol-DES mixture (choline chloride glycerol, 1:2), stirred at room temperature for 12 h, filtered, washed with water and ethanol, and dried at 80 °C to a constant weight. Before use, the SPE column containing 200 mg DES modified silica gel was prewashed with methanol (2.0 mL) and deionized water (2.0 mL), successively. Then an extraction solution containing ferulic acid (3.0 mL) was loaded onto the top of the column, and n-hexane (3.0 mL) was used as the carrier and methanol (3.0 mL) as the eluent. The results showed that the dynamic adsorption capacity of ferulic acid on the column was 0.033 mg/g, which was very close to the static adsorption capacity (0.034 mg/g); its recovery rate was higher on DES modified silica gel (89.7%) than that of blank silica gel (64.1%) [95].

#### Immobilized DESs in separation materials

Currently, most of DESs used have high viscosity, and their disadvantages such as easy wear and tear, leakage, and difficult recovery (due to their multiple components) also limit their large-scale industrial applications. To address these issues, immobilized DESs have also been developed and applied. Researchers use physical or chemical reactions to load DESs onto suitable solid materials (such as nanoparticles, graphene, cyclodextrin, metal organic frameworks, etc.). The resulting composite modified/bound by DESs not only possesses the characteristics of DESs, but also makes it more stable and easier to use. In addition, DESs containing polymerizable monomer components can also be prepared into polymers by polymerization, or DESs with gel factor structural characteristics can be self-assembled to obtain corresponding (hydro)gel. For possible large-scale applications, immobilization has the following significance:

- It effectively solves the problems of recovery and residue of DESs, making them more “green” and more conducive to their application in fields such as medicine, life science, and (functional) foods
- Immobilizing DESs onto high surface-area porous carriers (e.g., nanoparticles, graphene, MOFs) exploits the intrinsic porosity of the support to increase the accessible interfacial area for analyte–DES interactions, thereby improving contact and mass transfer. Such composites are sometimes termed ‘porous DES-based materials
- After use, recovery can be achieved through simple solid–liquid separation, which is convenient, simple, and does not require additional operations or reagents, thus avoiding cross contamination and reducing usage costs
- It can achieve continuous operation and improve production efficiency
- The performance of DESs in certain solvents can be improved by selecting appropriate DES loadings on solid phase carriers. For example, the solid phase extraction material of Fe_3_O_4_@GO-DES was obtained by immobilizing choline chloride-urea (1:2) on magnetic graphene oxide (Fe_3_O_4_@GO), which could be conveniently recovered for reuse by using external magnetic field.

Currently, researches in this field mainly focus on developing new SPE materials to achieve higher selectivity and processing capabilities. One representative of innovative research is DM-SPE. Compared to traditional SPE materials, it directly adds magnetic adsorbent to the sample solution, and sufficient dispersion is beneficial for the contact between the adsorbent particles and the analyte. After enrichment, post-processing is carried out using an external magnetic field. There was a report that the DESs of N-isopropylacrylamide-(3-acrylamidopropyl) trimethylammonium chloride (1:1) was used as a functional monomer, combined with graphite, ferric chloride, silica and other raw materials to obtain magnetic thermosensitive MIP. It could enhance the adsorption capacity of SPE material and has high reusability. After combined with high-performance liquid chromatography, rhein, triterpenoid saponins, aristolochic acid and other substances in Cassia seeds were accurately analysed [96]. By comparison, the preparation of non-magnetic DES-involved SEP materials is usually easier. For example, template molecule (chlorogenic acid, CA), functional monomer (acrylamide) and DESs (choline chloride-glycerol, 1:2) were dissolved in methanol–water and ultrasonicated, then crosslinking agent (ethylene glycol dimethacrylate) and initiator (2-methylpropionitrile) were added and polymerization occurred under heating. Rapid purification of CA from hawthorn extract was executed by SPE with the prepared DES-MIP material (see Fig. 6F), and the recovery of the established method for CA was 72.56%. Overall, DES-MIPs showed good purification characteristics in this study [97].

### EHS analysis and countermeasures

#### Greenness and biocompatibility

It is worth noting that using green solvents in a certain stage does not necessarily mean that the entire process is safe and friendly, and even whether green solvents are completely safe and friendly is still under continuous exploration. Chinese medicine products are closely related to health, so the large-scale use of a certain chemical medium must be carefully considered, requiring comprehensive research, sufficient data and scientific evaluation. Among numerous reports, DESs are non-toxic, environmentally friendly, and biodegradable benign solvents, especially those derived from natural compounds. Their impact on EHS is much smaller than that of IL as another famous green solvent, and they are more easily accepted and widely used. However, compared with IL, there are fewer reports on the toxicity and biodegradability of DESs in these aspects due to a late start. In limited literature, researchers have evaluated the risk of DESs (choline chloride-glycerol, choline chloride-ethylene glycol, choline chloride-Tri ethylene glycol and choline chloride-urea) using two Gram positive bacteria (*Bacillus subtilis* and *Staphylococcus aureus*), two Gram negative bacteria (*Escherichia coli* and *Pseudomonas aeruginosa*), and saltwater shrimp eggs [98]. The results showed that the four DESs had no inhibitory effect on the selected bacteria, but had cytotoxicity against saltwater shrimp eggs, and the DESs studied had higher cytotoxicity than their individual components. This may be due to property changes between individual components through hydrogen bonding, or it may result from high viscosity of DESs causing hypoxia or difficulty in movement of saltwater shrimp.

In an in vivo animal evaluation study, the natural DESs, (NADES) of betaine-glycerol was administered orally to rats for acute toxicity testing [99]. Each experimental group consisted of 6 rats, with a total of 2 groups. The rats were orally administered with 3 mL twice a day for 14 days. Eventually, 2 rats died, and the surviving animals exhibited reactions such as excessive water intake, reduced dietary intake, weight loss, hepatomegaly, and plasma oxidative stress. In addition, it has been found that there is transdermal absorption after contact with the skin, which is also the main pathway for non-volatile chemical media to enter the body. It has been concluded that DESs can bypass the barrier properties of the stratum corneum and enhance transdermal and paracellular transport by disrupting cell integrity, forming diffusion pathways in the stratum corneum, and extracting lipid components. Our group evaluated the transdermal penetration kinetics of eleven representative DESs using five kinetic models (zero‑order, first‑order, Higuchi, Hixson–Crowell, and Ritger–Peppas) [100]. The DESs studied included hydrophilic choline chloride‑based systems (e.g., ChCl:urea, ChCl:glycerol, ChCl:ethylene glycol) and carboxylic acid‑based DESs (e.g., ChCl:lactic acid, ChCl:malic acid), covering a range of HBD commonly used in extraction applications. The Ritger–Peppas model provided the best fit, indicating that diffusion through the stratum corneum was the rate‑limiting step. Spectroscopic and microscopic analyses confirmed that these DESs reversibly altered skin‑barrier integrity without causing persistent irritation, and the molar ratios of the DES components remained stable before and after application.

#### LCA of DES‑based TCM extraction

**Fig. 7:**
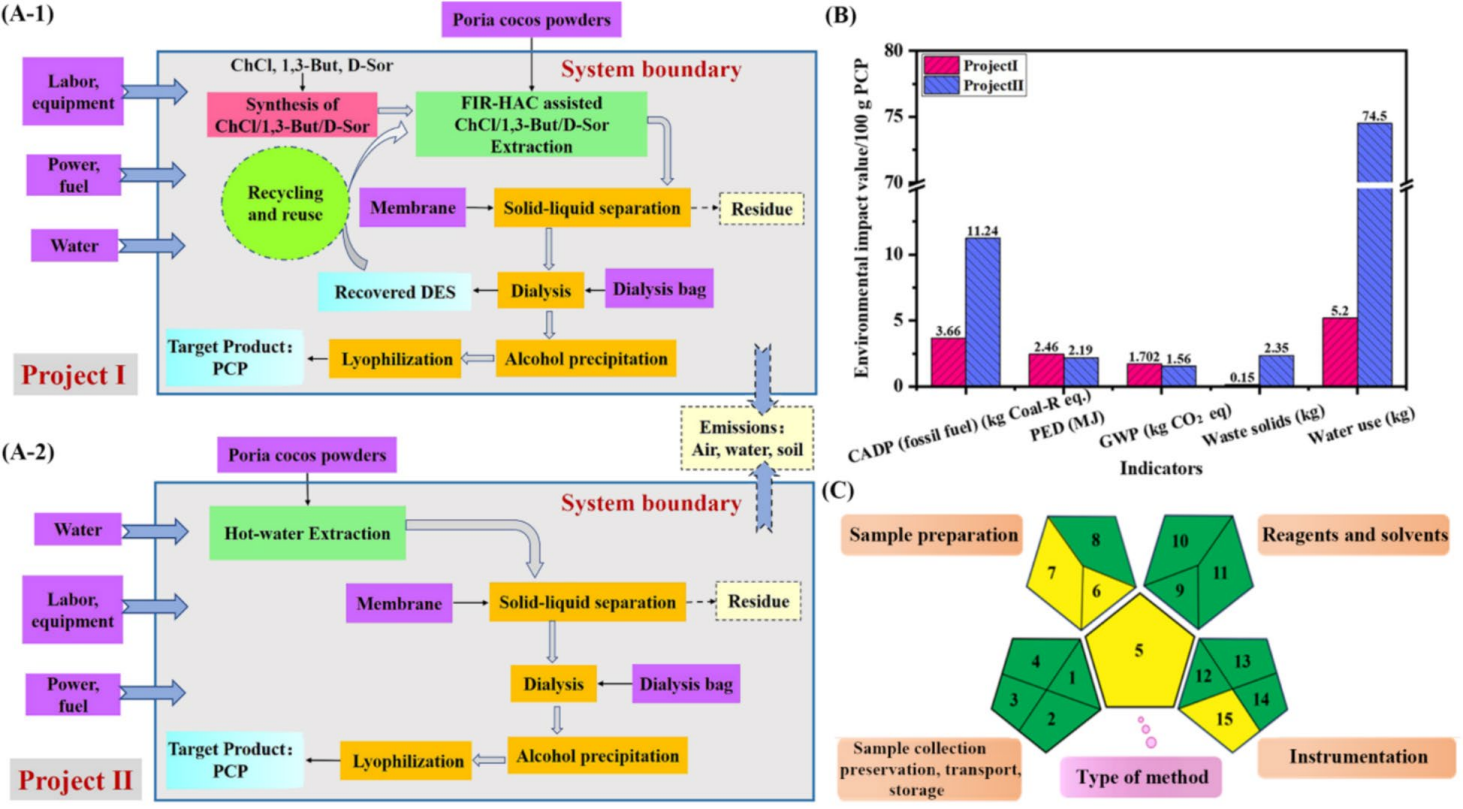
**A** LCA boundaries for FIR-HAC assisted DESs extraction process (Project I), traditional hot-water extraction process (Project II) of PCP (the process in the thick gray arrows represent the base flow); **B** Life cycle impact assessment (*LCIA*) with five indicators (fossil fuel depletion potential, CADP (fossil fuel); primary energy demand, *PED*; global warming potential, *GWP*; waste solids and water use). Results for environmental impact are given for 100 g PCP production chain based on Project I and Project II [73]; **C** GAPI assessment of the green profile of the evaluated procedures for DESs extraction process of bioactive fractions in *Paeoniae radix rubra* [102]

LCA is a standardized method for quantifying the environmental impacts of a product or process across its entire life cycle from raw material acquisition to disposal. In the context of DES‑mediated extraction of TCM compounds, LCA studies provide critical insights into the comparative sustainability of DES‑based methods versus conventional extraction techniques. A representative LCA study comparing a novel DES‑far‑infrared‑hot‑air‑circulation (FIR‑HAC) extraction of Poria cocos polysaccharides (Project I) with a conventional hot‑water extraction process (Project II) demonstrated significant environmental advantages for the DES‑based approach [73]. The system boundaries of both processes are illustrated in Fig. 7A-I-II. Key environmental impact indicators assessed included fossil‑fuel depletion potential (CADP), primary energy demand (PED), global warming potential (GWP), solid waste generation, and water consumption. The results, summarized in Fig. 7B, revealed that per 100 g of extracted polysaccharides, Project I achieved Fig. 7A-I, A 93.6% reduction in solid waste, A 69.7% decrease in fossil‑fuel depletion, Similar PED (2.46 MJ vs. 2.19 MJ) and GWP (1.70 kg CO₂‑eq vs. 1.56 kg CO₂‑eq) compared to Project II, Fig. 7A-II. The superior performance of the DES‑based process is largely attributed to the reusability of DES, which reduces solvent consumption across multiple extraction cycles, and the integration of energy‑efficient auxiliary techniques (FIR‑HAC) that lower thermal energy requirements. Another critical LCA observation is the strong influence of DESs composition on the overall environmental profile. DESs derived entirely from natural primary metabolites (NADES) typically exhibit lower human‑ and eco‑toxicity potentials than those containing synthetic quaternary ammonium salts or metal ions. Therefore, selecting bio‑based, low‑toxicity HBD and HBA such as choline chloride combined with organic acids, sugars, or amino acids can significantly enhance the green credentials of the extraction process.

In summary, LCA provides a valuable tool for optimizing DES‑based TCM extraction systems not only for efficiency and selectivity but also for minimal environmental footprint. Future research should incorporate LCA at an early stage of process design to identify trade‑offs, guide solvent selection, and ensure that the green potential of DESs is fully realized in sustainable TCM production.

#### Green metric assessment tools for DES‑based extraction

The reported green criteria tools include the National Environmental Method Index (NEMI), Analytical Eco-Scale, hexagon-CALIFICAMET, Analytical GREEnness Metric approach (AGREE) Analytical Greenness Metric for Sample Preparation (AGREEprep), Analytical Ecological scale (AES), RGB (Red Green Blue), White Analytical Chemistry (WAC) models, Sample Preparation Metric of Sustainability (SPMS), green Analytical Procedure Index (GAPI), Complementary Green Analytical Procedure Index (ComplexGAPI), Modified GAPI (MoGAPI) and Complex Modified GAPI (ComplexMoGAPI), etc. Many of them are easy for Chinese medicine technicians to master; the process is not complicated and the data are easy to obtain. Especially, their combinational application is a comprehensive evaluation strategy. Among them, GAPI provides a unique assessment way of the entire method and provides more information [101]. It mainly includes the steps of sample collection, sample preparation, and final analysis, which uses pictograms of five pentagons to visualize the greenness of various programs in the form of a graph, in which the green, yellow, and red colors express the low, medium, and high levels of greenness, respectively. Figure 7C shows the GAPI assessment of the green profile of the evaluated procedures for DES extraction process of bioactive fractions in Paeoniae radix rubra developed by Chang’s group [102]. which is mainly composed of green and yellow blocks. It can indicate that their method was friendly and had little impact on the environment.

### SWOT analysis on DES applications for Chinese medicines

#### Strengths

DESs have shown significant advantages in the field of extraction and separation of TCM, making them a powerful alternative to traditional organic solvents. These advantages are not only reflected in environmental friendliness, but also in its excellent extraction efficiency and wide applicability. The green and environmentally friendly characteristics are the most highly regarded core advantages of DESs. Compared with traditional organic solvents such as methanol and acetone, DESs have lower ecological risks. From the perspective of life cycle assessment, DESs have been reported to exhibit a carbon footprint up to 40% lower than that of typical IL when considering raw material sourcing, synthesis, and end‑of‑life disposal [73, 103]. This environmentally friendly feature makes DESs particularly suitable for fields such as food and medicine that have strict requirements for solvent residues.

The designability and structural diversity of DESs provide infinite possibilities for their application in complex separation tasks. By selecting different HBA and HBD, researchers can precisely regulate the polarity, viscosity, and selectivity of DESs, forming “customized solvents” for specific target compounds. In recent years, the development of ternary DESs has further enhanced this designability by introducing a third component. The high adjustability enables DESs to adapt to the extraction and separation needs of various compounds, ranging from polar to non-polar, from small molecules to large molecules, and from hydrophilic to hydrophobic.

The outstanding performance in extraction efficiency and selectivity is a key factor for the rapid development of DESs. DESs often achieve higher extraction rates and better selectivity than traditional solvents through various interactions such as hydrogen bonding networks and π-π stacking. DESs also demonstrate good process compatibility and can work in conjunction with various enhanced extraction techniques such as ultrasound and microwave to further improve efficiency. This compatibility enables DESs to be easily integrated into existing extraction devices, reducing the technological threshold for industrial conversion.

#### Weaknesses

The issue of high viscosity is one of the most prominent challenges faced by DESs. Many DESs, especially those based on choline chloride and polycarboxylates, exhibit high viscosity at room temperature, which seriously affects the mass transfer rate and mixing efficiency. Although introducing a third component can increase the viscosity of certain DESs or reduce viscosity through heating, these methods often come at the cost of sacrificing energy efficiency or increasing process complexity.

The lack of standardization system constitutes another major obstacle to the research and application of DESs. At present, there is a lack of unified synthesis standards, purity standards, and performance evaluation methods in the DESs field. Different research teams use different preparation processes and characterization methods, making it difficult to directly compare and replicate research results. For example, water content has a significant impact on the properties of DESs (a small amount of water can reduce viscosity, but too much can damage the hydrogen bond network), but different studies have defined the water content of “water containing DESs” differently. Similarly, there is a lack of standardized testing protocols for the recovery rate and reuse frequency of DESs, and some studies may have reported deviations in recovery rates due to different operating methods. This lack of standardization not only increases the difficulty of technology transfer, but also delays the process of DESs moving from laboratories to industrialization.

Cost and scale challenges are also issues that cannot be ignored in the practical application of DESs. Although the raw materials of DESs, such as choline, urea, organic acids, etc., are relatively low in price, the cost of high-purity reagents is still considerable, especially for pharmaceutical and food grade applications. Most DESs research is still at the small-scale laboratory stage (from milliliters to upgrades), and engineering problems such as mass transfer, heat transfer, and phase separation may be encountered during the scaling up process. From optimizing laboratory conditions to stable operation in industrial production, DESs technology still needs to overcome many engineering scaling challenges.

The controversy over biodegradability is a potential threat to the reputation of DESs as “green solvents”. Although DESs are often promoted as biodegradable and environmentally friendly alternatives, increasing research suggests that the ecological toxicity and degradability of certain DESs components may be underestimated. Especially those DESs containing quaternary ammonium salts (such as choline derivatives) or aromatic compounds. Not all DESs are as environmentally friendly as initially claimed. This uncertainty may lead to future regulatory restrictions, especially in areas governed by strict environmental regulations. Finally, the imperfect separation and recycling technology also limits the recycling and economic viability of DESs. Although DESs can theoretically be recycled and reused, they often face problems such as product residue, decreased solvent purity, and performance degradation in practical operations.

#### Opportunities

The green chemistry policy has created a favorable macro environment for the promotion and application of DESs. The increasingly strict environmental regulations and solvent usage restrictions worldwide are forcing various industries to seek alternatives to traditional harmful solvents. The EU REACH regulation, the US EPA’s green chemistry program, and China's “dual carbon” strategy are all driving the adoption of more sustainable chemical processes in the industry. In this context, DESs, as a solvent system that complies with green chemistry principles, are expected to receive policy support and market favor. Especially in the pharmaceutical and food industries, as consumer demand for “clean labels” and “green manufacturing” grows, the use of natural ingredients extracted from DESs may gain a premium advantage.

Secondly, the high-value utilization of TCM waste provides a broad application space for DESs. These raw materials often contain valuable bioactive ingredients, but traditional methods are difficult to extract economically and effectively. DESs, with their designability and gentle extraction characteristics, are becoming a powerful tool for the value-added transformation of natural waste resources. At the same time, the rise of computer-aided design and artificial intelligence has brought revolutionary opportunities for DESs development. Traditional DESs screening relies on trial-and-error methods, which are time-consuming, labor-intensive, and costly. Nowadays, quantum chemistry computing and machine learning are accelerating the rational DESs design process. With the improvement of computing power and algorithm advancement, it is expected to establish more accurate DESs structure–property, structure–activity, and structure–toxicity relationship models in the future, achieve virtual screening and performance prediction, and significantly reduce experimental development costs.

Finally, emerging markets and special application demands also provide differentiated development opportunities for DESs. In high value-added fields such as biomedicine and functional foods, the special requirements for solvent systems (such as high selectivity, mild conditions, low residue, etc.) perfectly match the advantages of DESs. Although the market size may be smaller than that of bulk chemicals, the profit margin and technical barriers are higher, which is conducive to the commercialization of DESs applications.

#### Threats

Although DESs have broad prospects in the field of TCM extraction and separation, their development still faces threats from various aspects such as technological competition, economic challenges, knowledge barriers, and regulatory uncertainty. These external factors may delay or even hinder the widespread application and commercialization of DESs. Alternative technology competition is the most direct threat faced by DESs. In the field of green solvents, DESs are not the only choice. Alternative technologies such as supercritical fluids (such as CO_2_), IL, and bio-based solvents have their own advantages, and some have been industrialized. Especially, bio-based solvents such as ethyl lactate and 2-methyltetrahydrofuran, have the advantages of greenness and low cost, and some of their properties overlap with DESs and may divert market demand. Overall, the threats faced by DESs are multidimensional, including direct competition from alternative technologies and constraints from insufficient technological maturity. In the future, it is necessary to optimize solvent performance through computer-aided design, develop low-cost recycling processes, and promote the establishment of industry standards to address these challenges.

## Conclusions

The integration of DESs with TCM extraction and separation represents a promising and rapidly evolving frontier in green chemistry. DESs offer tunable physicochemical properties, enhanced solvation capability, improved selectivity, and reduced environmental impact compared to conventional organic solvents. Their compatibility with various extraction techniques such as ultrasound, microwave, mechanochemical, and aqueous biphasic systems further underscores their versatility and potential for sustainable process development. However, it is essential to recognize that DESs are not a universal solution and come with notable limitations. Challenges such as high viscosity, limited scalability, incomplete toxicological and biodegradability data, and the potential for reduced extraction efficiency in systems where DESs components are strongly self-associated must be rigorously addressed. Furthermore, the influence of water on DESs microstructure in aqueous systems and the need for tailored solvent design for specific herbal matrices highlight the importance of rational, case-by-case optimization.

Future advancements in DES technology will depend on interdisciplinary collaboration, supported by computational modeling, machine learning, and LCA tools to guide solvent design, process optimization, and environmental impact evaluation. Policy support and industrial engagement will also be crucial to translate laboratory innovations into scalable, economically viable, and environmentally responsible practices.

In summary, while DESs hold significant promise for modernizing TCM extraction in alignment with green chemistry principles, their successful implementation requires a balanced approach that acknowledges both their strengths and existing challenges. Continued research aimed at overcoming these limitations will be essential to fully realize the potential of DESs in enabling safer, more efficient, and sustainable development of TCM.

## Data Availability

No datasets were generated or analysed during the current study.

## Abbreviations

ABS: Aqueous biphasic systems
AEP: Aquatic ecotoxicity potential
AES: Analytical Ecological scale
AGREE: Analytical GREEnness Metric approach
AGREEprep: Analytical Greenness Metric for Sample Preparation
AI: Artificial intelligence
AMP: Astragalus polysaccharides
ANN: Artificial neural network
AP: Acidification potential
CA: Chlorogenic acid
CEP: Cepharanthine
ChCl/Glu: Choline chloride-glucose
COSMO-RS: Conductor-like screening model for real solvents
ComplexGAPI: Complementary Green Analytical Procedure Index
ComplexMoGAPI: Complex Modified GAPI
CP: *Codonopsis pilosula*
DES: Deep Eutectic Solvent
DES-FIR-HACE: Deep Eutectic Solvent-Far Infrared Radiation-Hot Air Circulation Extraction
DFT: Density Functional Theory
DTAC: Dodecyl Trimethyl Ammonium Chloride
EHS: Environmental-Health-Safety
EP: Eutrophication Potential
FTIR: Fourier Transfer Infrared Spectroscopy
GAC: Green Analytical Chemistry
GAPI: Green Analytical Procedure Index
GO: Graphene oxide
GWP: Global warming potential
HA: Hexanoic acid
HBAs: Hydrogen bond acceptors
HBDs: Hydrogen bond donors
HFIP: Hexafluoroisopropanol
HSCCC: High speed counter current chromatography
HTP: Human toxicity potential
LCA: Life cycle assessment
LLE: Liquid–liquid extraction
MAE: Microwave-assisted extraction
MD: Molecular dynamics
Men/Lac: D,L-Menthol-D,L-Lactic acid
MIP: Molecularly imprinted polymers
ML: Machine learning
MoGAPI: Modified GAPI
NADES: Natural deep eutectic solvent
NEMI: National Environmental Method Index
NPCE: Negative pressure cavitation extraction
ODP: Ozone layer depletion
PCP: *Poria cocos* Polysaccharides
PEG: Polyethylene glycol
PEEK: Polyetheretherketone
POCP: Photochemical oxidation potential
QC: Quantum chemical
QM: Quantum mechanics
QSAR: Quantitative structure–activity relationship
R&D: Research and development
RGB: Red green blue
SPE: Solid phase extraction
SPMS: Sample Preparation Metric of Sustainability
SWOT: Strength-weakness-opportunity-threat
TBAC: Tetrabutylammonium chloride
TCM: Traditional Chinese medicines
TEP: Terrestrial ecotoxicity potential
TLC: Thin layer chromatography
TOMAC: Methyltrioctylammonium chloride
UAE: Ultrasound-assisted extraction
UNIFAC: Universal quasi-chemical functional-group activity coefficient models
WAC: White Analytical Chemistry
